# Conventional and Immunodiagnostic Platforms for the Detection of Major Mycotoxigenic Fungi and Their Toxins for Food Safety

**DOI:** 10.3390/foods15203674

**Published:** 2026-10-16

**Authors:** Dragana Bartolić, Mira Stanković, Miodrag Mićić, Slavica Stanković, Dunja Banđur, Jelena Grebenar, Ksenija Radotić

**Affiliations:** 1 Institute of Multidisciplinary Research, National Institute of the Republic of Serbia, University of Belgrade, Kneza Viseslava 1, 11030 Belgrade, Serbia; mira.mutavdzic@imsi.bg.ac.rs (M.S.); dunja.bandjur@imsi.bg.ac.rs (D.B.); 2 Department of Engineering Design Technology, Cerritos College, 11110 Alondra Blvd, Norwalk, CA 90650, USA; mmicic@cerritos.edu; 3 Maize Research Institute, Slobodana Bajića 1, 11185 Belgrade, Serbia; sstojkov@mrizp.rs; 4 Faculty of Chemistry, University of Belgrade, Studentski trg 12-16, 11158 Belgrade, Serbia; jelenagrebenar18@gmail.com

**Keywords:** mycotoxigenic fungi, mycotoxins, molecular diagnostics, sample preparation, point-of-need testing, ELISA, Lateral-flow assays, immunosensors, immunodiagnostics

## Abstract

Mycotoxigenic fungi contaminate cereals, nuts, fruits, and other food commodities, threatening human and animal health. This review focuses on *Aspergillus flavus*, *Fusarium verticillioides*, and *Alternaria alternata* as representative mycotoxigenic fungi because of their importance as food contaminants and their production of major food-relevant mycotoxins. Some methods detect fungal presence or toxigenic potential, whereas others directly quantify toxins. Fungal identification or detection of biosynthetic genes indicates presence or toxin-producing potential, respectively, but neither replaces direct mycotoxin measurement. The diversity of targets, toxins, and food matrices remains a challenge for accurate and practical analysis. Particular attention is given to immunodiagnostic approaches, including enzyme-linked immunosorbent assays (ELISAs), as well as complementary formats such as lateral flow immunoassays (LFIAs), and immunosensors, for laboratory and on-site food safety monitoring. The advantages and limitations of these approaches, with emphasis on sensitivity, specificity, matrix effects, and sample preparation, are discussed. Future aspects will focus on integrated, matrix-validated workflows combining robust sample preparation, multiplex recognition, portable readout, and appropriate quality controls to enable faster and more reliable monitoring of fungal contamination and mycotoxins throughout the food and feed chain.

## 1. Introduction

**Figure 1:**
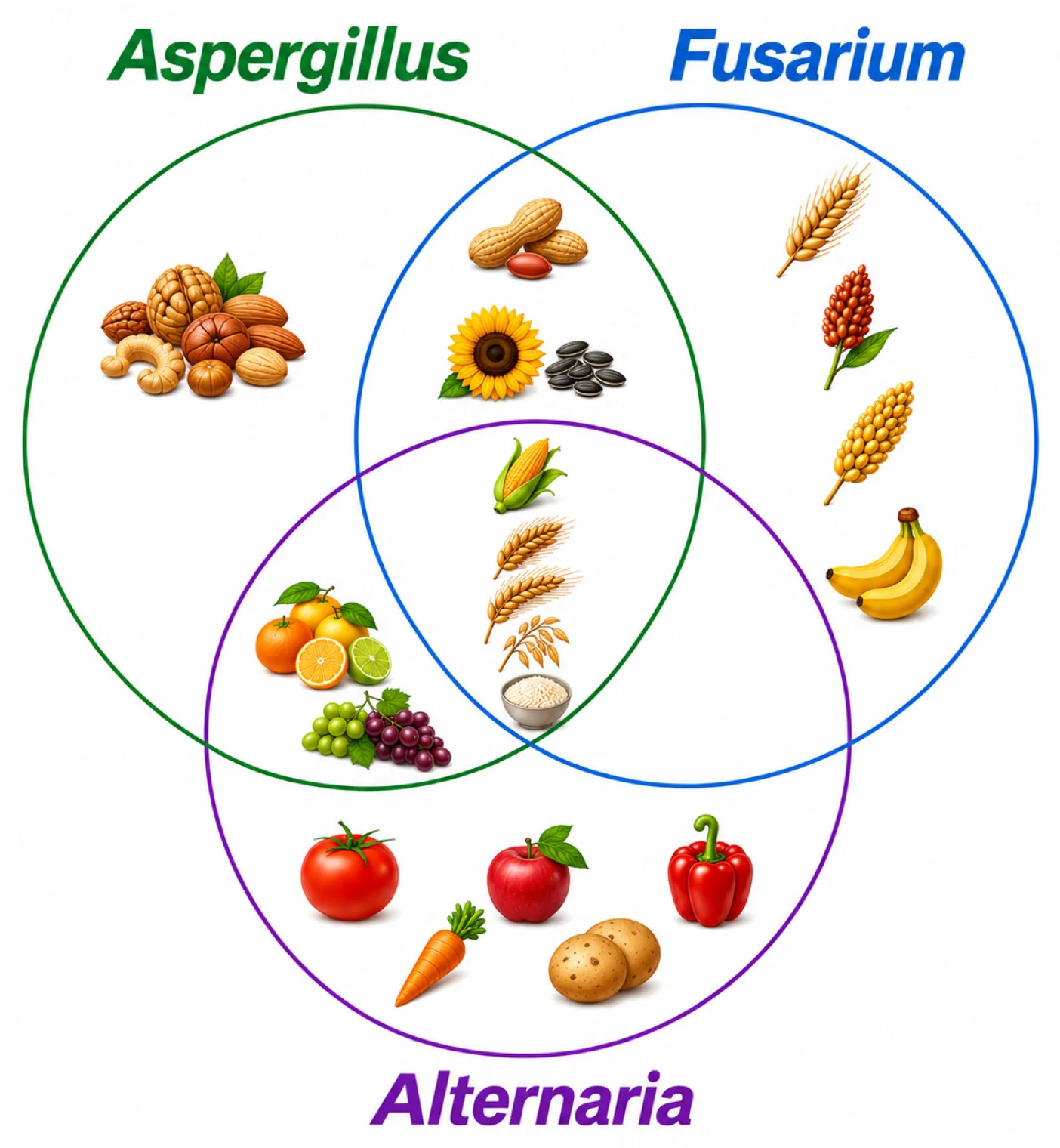
Major food commodities associated with mycotoxigenic species of the genera *Aspergillus*, *Fusarium*, and *Alternaria*.

Mycotoxigenic fungi are a major threat to global food safety, with their diversity and invasion potential expected to increase under future climate scenarios [1,2,3]. These fungi produced toxic secondary metabolites known as mycotoxins during the crop growth and storage, which can subsequently enter the human food chain through livestock fed on contaminated feed [2,3,4]. The genera *Aspergillus*, *Fusarium*, and *Alternaria* represent the most predominant mycotoxigenic fungi in agriculture, as they simultaneously compromise crop yields and pose severe risks to human and animal health [2,3,4]. In agricultural commodities, multiple fungal species may occur simultaneously, potentially resulting in the production of different classes of mycotoxins and posing challenges for food/feed safety and potential risks to consumers [3,5]. Cereals are vulnerable to all three fungal genera, whereas oilseeds, nuts, fruits, and vegetables exhibit genus-specific susceptibility, as shown in Figure 1.

The genus *Aspergillus* contains several species associated with the contamination of agricultural commodities, including maize, cottonseed, peanuts, tree nuts, fruits, and other food products [3,6]. The *Aspergillus* section *Flavi* includes important aflatoxin-producing species, among which *A. flavus* is a major producer of aflatoxins (AFs), particularly aflatoxin B1 (AFB1), and frequently contaminates commodities such as cottonseed, maize, peanuts, and tree nuts during both pre- and post-harvest stages [5,6]. Besides, AFs have been reported in a wide range of food commodities, including fresh berries, pome fruits, dried figs, juices, and wine [7]. AFB1 is the most potent of the major naturally occurring aflatoxins and is classified as a Group 1 human carcinogen by the International Agency for Research on Cancer (IARC) [8]. Other *Aspergillus* species, particularly those associated with stored products, can also contribute to the occurrence of other important mycotoxins, including ochratoxin A (OTA), which is relevant to the safety of cereals, dried fruits, coffee, wine, and other food commodities [9,10]. *Fusarium* species are among the most important mycotoxigenic fungi associated with cereals, particularly maize and wheat, and can produce several chemically and toxicologically distinct groups of mycotoxins. *F. verticillioides* and *F. proliferatum* are major producers of fumonisins (FUM), particularly fumonisin B1 (FB1) and fumonisin B2 (FB2), and frequently colonize maize [2,11]. *F. graminearum* is an important producer of deoxynivalenol (DON) and zearalenone (ZEN), with DON being a major trichothecene associated with cereal contamination and ZEN exhibiting estrogenic activity [2,12]. Other *Fusarium* species can produce type A trichothecenes, including T-2 and HT-2 toxins, which are also important contaminants of cereals and cereal-based foods. The co-occurrence of several *Fusarium* species may therefore result in the simultaneous presence of different mycotoxin classes in the same agricultural commodity, complicating both exposure assessment and analytical monitoring [3]. *Fusarium* diseases may cause substantial losses of cereals and other crops [7,13], but from a food-safety perspective, the major concern is the contamination of harvested commodities with *Fusarium* mycotoxins and their subsequent transfer into the food and feed chains. *Alternaria* species frequently occur in fruits, vegetables, cereals, and other plant-derived foods and can produce several mycotoxins, including alternariol (AOH), alternariol monomethyl ether (AME), and tenuazonic acid (TeA) [4,14,15,16,17,18]. The presence of *Alternaria* in food commodities is of particular concern because toxin production may occur during both crop growth and storage [19,20,21,22]. Unlike several major *Fusarium* and *Aspergillus* mycotoxins, major *Alternaria* toxins are currently considered emerging mycotoxins and remain without harmonized maximum levels under EU legislation, despite increasing evidence of their occurrence in food and potential health risks [23]. This highlights the need for continued monitoring of *Alternaria* toxins in food commodities.

Fungal growth does not necessarily indicate mycotoxin contamination, as toxin biosynthesis depends on specific environmental and physiological conditions [24]. Conversely, mycotoxins may persist in food and feed after fungal growth has ceased and may undergo chemical or biological transformation during processing, storage, and digestion. In addition to their occurrence as parent compounds, mycotoxins may therefore be present as modified or masked forms, in which the toxin structure is altered through plant, fungal, or processing-related mechanisms [25,26]. These modified forms may not always be detected by analytical methods targeting the parent mycotoxin but can potentially contribute to overall exposure when released or metabolized in the digestive tract [27]. Consequently, consideration of both parent and modified mycotoxins is increasingly important for comprehensive food-safety assessment.

Mycotoxigenic fungi and their toxins have been linked to a wide range of adverse health effects, including acute toxicity, mutagenicity, carcinogenicity, genotoxicity, teratogenicity, immunosuppression, hormonal, gastrointestinal and renal disorders, as well as allergic reactions [2,16,28]. The nature and severity of these effects vary considerably among fungal species and according to the specific mycotoxins produced [24]. The simultaneous occurrence of multiple mycotoxins may further complicate risk assessment because consumers can be exposed to mixtures rather than to individual toxins. Therefore, reliable detection of both mycotoxigenic fungi and their associated mycotoxins is an essential component of food-safety monitoring.

The widespread occurrence of mycotoxins in food commodities, together with their adverse health effects, has led to increasingly stringent food-safety regulations. In the European Union (EU), Regulation (EU) 2023/915 established maximum levels for major mycotoxins, including AFs and OTA, as well as *Fusarium*-related DON, ZEN, FUM and T-2/HT-2 toxins, with subsequent amendments further revising some limits [9,10]. Major *Alternaria* toxins, however, currently remain without harmonized maximum levels under EU legislation, despite increasing evidence of their occurrence in food and potential health risks [23]. AFs and FUM are already subject to strict regulatory limits [29], whereas toxins produced by *A. alternata*, including alternariol, are considered emerging mycotoxins and remain under increased scientific and regulatory surveillance [23]. These regulatory developments emphasize the need for reliable, sensitive, and rapid analytical methods for monitoring mycotoxigenic fungi and their toxins in food [30,31].

In this review, we provide a critical overview of conventional and emerging affinity -based approaches for the detection of toxigenic fungi and their mycotoxins, with particular emphasis on immunodiagnostic platforms. We first discuss established culture-microscopy-, and molecular-based approaches, followed by antibody- and aptamer-based assays, and immunosensors. Particular attention is given to recent advances in nanomaterial-enhanced detection, multiplex and multimodal platforms, and artificial intelligence (AI)-supported interpretation. The major challenges and future perspectives related to analytical performance, sample preparation, matrix effects, stability, standardization, field validation, and regulatory acceptance are discussed. We further consider the potential for simultaneous detection of toxigenic fungi and mycotoxins and the transition toward rapid, portable, and point-of-need testing across the agricultural and food production chains. 

## 2. Conventional Platforms for Detection of Mycotoxigenic Fungi and Their Toxins

Culture establishes recovery of viable fungal propagules under the selected conditions. Polymerase chain reaction (PCR) detects fungal DNA, using broad identification markers such as ITS1/ITS2 or more specific targets selected according to the fungal group. Biosynthetic gene detection indicates genetic potential, not demonstrated toxin production [32,33,34]. Chromatographic methods determine the toxins themselves when suitable analyte standards and validation are available [35]. Spectral screening may instead detect correlated changes in the commodity. Fungal isolation does not prove toxin contamination, and toxin detection does not prove that a viable producer remains.

### 2.1. Phenotypic Identification

Phenotypic identification may integrate colony color and texture, growth rate, sporulation, microscopic morphology, temperature tolerance, water-activity preference, substrate utilization, and pathogenic behavior. Modern fungal taxonomy uses these observations within a polyphasic framework that also incorporates reference cultures, ecology, type material, and sequence data [36,37,38]. In food mycology, culture-based enumeration generally begins with representative collection, homogenization or blending, serial dilution, inoculation onto selective or differential media, incubation, and colony counting [39]. The United States Food and Drug Administration Bacteriological Analytical Manual recommends analysis of 25 to 50 g portions, with larger laboratory samples when greater reproducibility is needed, followed by homogenization in peptone water and replicate surface plating. Dichloran rose Bengal chloramphenicol agar is commonly used for foods with water activity above approximately 0.95, whereas dichloran 18% glycerol agar is preferred for drier commodities containing xerophilic fungi. Plates are ordinarily incubated near 25 °C and counted after 5 days, with an additional 48 h when growth is absent or insufficient [39]. Culture-based methods remain an important standard for fungal isolation and identification due to their simplicity, reliability, and minimal equipment requirements [40,41]. Detection of mycotoxigenic fungi in crops and food commodities relies on visual examination, culturing on artificial media, and morphological identification. Examination of colony characteristics and reproductive structures can identify fungal taxa associated with mycotoxin production, providing an early indication of potential mycotoxin contamination in food and feed [42].

**Figure 2:**
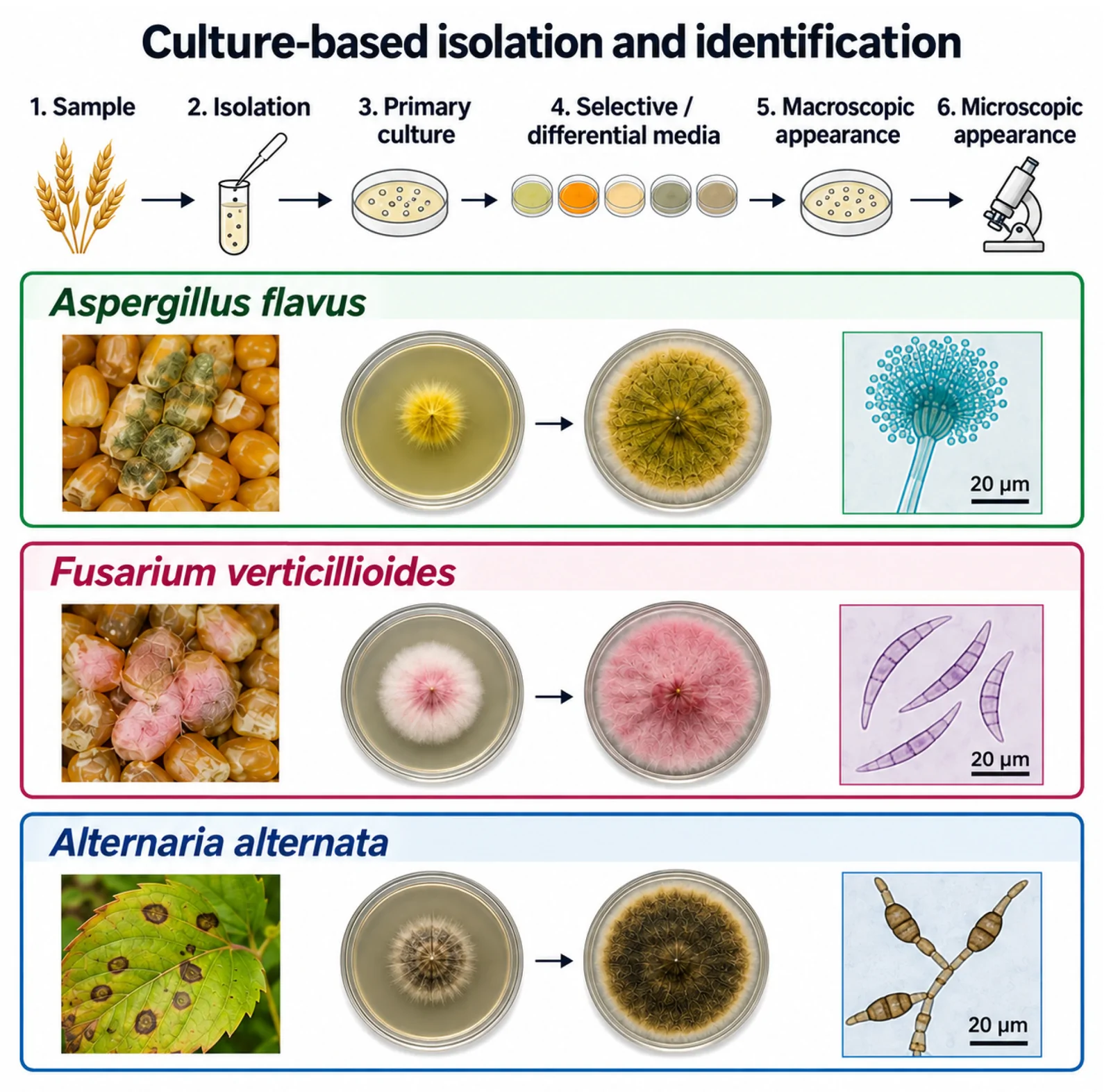
Illustrative workflow for culture-based isolation and phenotypic identification of three mycotoxigenic fungi: *A. flavus*, *F. verticillioides*, and *A. alternata* on different media at 25–28 °C after 3 and 7 days of incubation.

A viable propagule placed on a suitable medium germinates, grows, and forms a visible colony that can be counted, isolated, archived, and tested further. Potato dextrose agar (PDA) is one of the most commonly utilized media for fungal cultivation, but the optimal choice depends on the specific target fungal species [43]. Figure 2 shows the isolation and cultivation of three representative mycotoxigenic fungi, highlighting their distinct phenotypic differences in colony color across different selective media. It has been reported that *A. flavus* on PDA forms olive to dark green conidia within three days. Colonies exhibit a flat white margin with an elevated, rugose center, and certain isolates produce dark, globose sclerotia [44,45,46]. Diba et al. (2007) demonstrated that using differential media is a reliable and sensitive morphological method for identifying *Aspergillus* species, with *A. flavus* emerging as the dominant isolate [47]. Appropriate media enables distinguishing specific morphotypes, although culture conditions can also influence their appearance [47]. On PDA, *A. alternata* formed dark olive-brown to black colonies with dense mycelium, abundant sporulation, and irregular margins [19], whereas *F. verticillioides* initially produced white, floccose mycelium that became reddish with age, with pigmentation ranging from absent to violet or dark violet [48].

Direct microscopy detects fungal structures, and can provide preliminary assessment of obligate biotrophs like rust and powdery mildew fungi that cannot be routinely cultured [38,49].

The major strength of culture is the biological information preserved in a viable isolate. An isolate can be sequenced, archived, tested for pathogenicity, examined for fungicide susceptibility, evaluated for toxin-producing capacity, and used in epidemiological studies. Culture is also comparatively open-ended because it can recover unexpected cultivable organisms that a target-specific primer, probe, or antibody (Ab) would miss. These advantages are balanced by substantial limitations. Colony counts depend on medium composition, oxygen, water activity, incubation temperature and duration, physiological injury, dormancy, and competition. One colony may originate from a single spore, a clump of propagules, or a hyphal fragment, so a colony-forming unit is not a direct cell or spore count. Rapidly spreading fungi can obscure slower colonies, and a negative culture means only that no organism was recovered under the chosen conditions [50,51]. Morphological identification requires substantial training and experience. Interpretation can be subjective when characters are ambiguous or closely related species overlap. Phenotypic variation, mixed cultures, poor sporulation, and cryptic species further complicate identification [39,49]. Colony and microscopic morphology remain valuable for preliminary placement, but confident species-level identification increasingly requires integration of morphology, physiology, and phylogenetic evidence [37]. Isolation of a potentially toxigenic species does not establish that the sampled commodity contains its toxin, and toxin detection does not prove that a viable producer remains present. Traditional pathogen identification methods have limited repeatability because results vary significantly depending on the substrate, growth conditions, light, and temperature [52]. Basic culture requires modest instrumentation, although confirmatory identification adds equipment and analyst time. Incubation also delays results relative to many direct assays [39,53].

### 2.2. Conventional and Real-Time PCR

Morphological identification alone is often insufficient for species-level differentiation, given that closely related taxa often exhibit overlapping macro- and micro-morphological features [52]. PCR can detect fungal DNA before visible growth when extraction and target selection are appropriate [32,33,54]. PCR sensitivity in food matrices depends strongly on sampling, DNA extraction efficiency, fungal biomass, PCR inhibitors, and assay validation [54]. Ordinary DNA detection does not distinguish viable from non-viable fungal material [32,34]. These methods have the advantages that the structure of DNA remains stable with physiological stages, and its composition does not depend on culture conditions. DNA regions differ in variability allowing assays directed at species or broader taxonomic groups. Reliable identification requires primers validated for the intended species or target group [55,56]. No single primer set should be assumed suitable for every genus considered here. Closely related taxa should be included when testing specificity [56,57,58]. The genetic markers used with this purpose are the internal transcribed spacers, ITS1 and ITS2 [56,59], and protein-coding genes such as glyceraldehyde-3-phosphate dehydrogenase (*gpd*) and *Alt a 1* [57]. The *Alt a 1* gene encodes an *Alternaria* allergen whose homologues exist in related taxa [57,58]. Marker selection must therefore account for the intended taxonomic resolution. Conventional PCR, multiplex PCR, quantitative real-time PCR, and digital PCR have been successfully applied for detecting and quantifying fungal DNA in food matrices [32,33,34,54,60]. Conventional PCR is used to identify mycotoxigenic *Aspergillus* [41], *Fusarium* [11,61,62], *Alternaria* [63] species, while multiplex PCR allows for their simultaneous detection in a single reaction. Targeting mycotoxin biosynthetic genes indicates genetic potential [33,34,60].

It does not demonstrate gene expression or quantify toxin in the sampled food. PCR performance depends on extraction efficiency and inhibition controls, as well as validated primers [36,40]. Equipment and staffing requirements vary between conventional and automated workflows. Its suitability for routine screening therefore depends on the analytical purpose, rather than an inherent throughput disadvantage. PCR complements immunoassays but cannot confirm a toxin result merely by detecting fungal DNA.

### 2.3. Chromatographic and Spectroscopic Techniques

Chromatographic techniques are among the most commonly employed methods for mycotoxin analysis in agricultural products. Thin-layer chromatography (TLC) provides a rapid visual tool for mycotoxin identification, while high-performance liquid chromatography (HPLC) and ultra-high-performance liquid chromatography (UHPLC) offer improved resolution and faster analysis. These techniques can be coupled with different types of detectors, including fluorescence, ultraviolet (UV), diode-array (DAD), and mass spectrometry (MS) detectors [63]. Mycotoxin analysis requires very low detection limits (LODs), typically in the µg kg^−1^ (ppb) range [35,63]. While UV and fluorescence detectors have traditionally been used for chromatographic detection of mycotoxins, the development of LC-MS and LC-MS/MS methods has expanded the analytical capabilities for mycotoxin detection, offering high sensitivity and selectivity that can vary depending on the analytical method, instrumentation, matrix, and target mycotoxin [35]. LC-MS/MS is the method of choice for the simultaneous determination of multiple mycotoxins [22]. The current analytical techniques are time consuming, expensive and special equipment are required [17,64]. However, these methods remain essential for regulatory compliance and reference testing [63].

Ultraviolet-visible (UV-Vis), fluorescence, Fourier transform infrared (FTIR), and near-infrared (NIR) spectroscopy provide complementary optical information [58,61,65,66]. Raman spectroscopy measures vibrational features and can also support analytical recognition [67]. Direct toxin measurement must be distinguished from spectral associations with infection or commodity composition. For example, fluorescence signa-tures in contaminated maize may contain contributions from both toxins and the sur-rounding matrix [68,69]. Multivariate analysis can separate useful spectral variation, but it does not establish analyte identity by itself. Calibration against suitable reference measurements and independent matrix validation remain necessary [67,68,69].

## 3. Immunodiagnostic Platforms for Detection of Mycotoxigenic Fungi and Their Toxins

**Figure 3:**
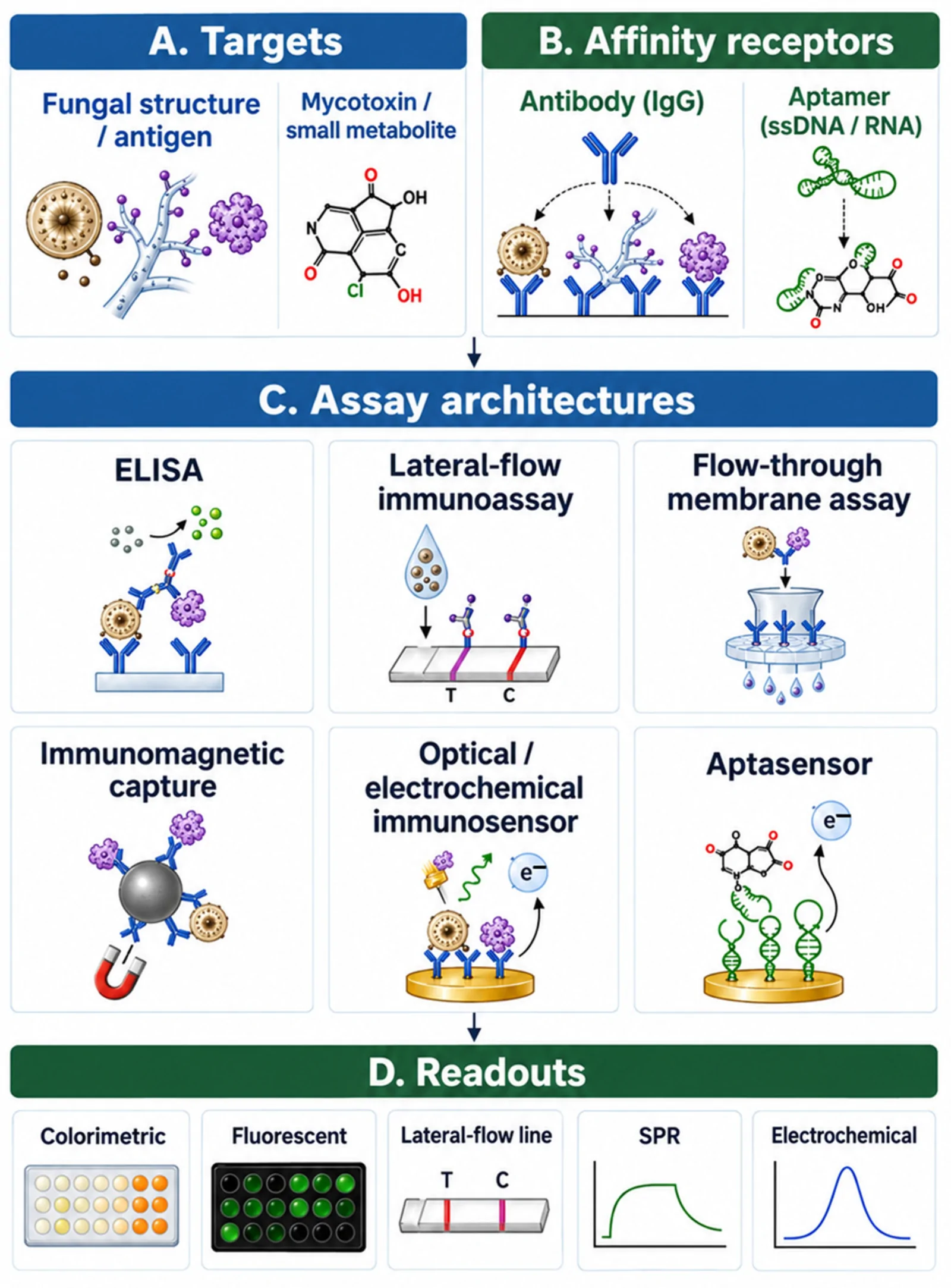
Affinity-based detection of fungal structures (spores and hyphae), soluble fungal antigens, and mycotoxins (**A**) using different affinity ligands (**B**), which can be incorporated into various assay formats (**C**), including ELISA, lateral-flow, flow-through, immunomagnetic, and immunosensor platforms, with optical, magnetic, or electrochemical readouts (**D**).

Immunodiagnostic detection platforms detect fungal structure or products through selective molecular recognition (Figure 3). Fungal detection relies on the recognition of fungal antigens or other specific structural components, whereas mycotoxins, as small molecules, are generally detected using competitive immunoassay formats. Enzyme-linked immunosorbent assays (ELISAs) and lateral flow immunoassays (LFIAs) are widely used for mycotoxin screening [70,71]. Immunosensors offer additional formats, although portability and validation depend on the individual system [70,72]. The use of immunological methods for detecting specific microorganisms provides a rapid and simple approach, with accuracy largely dependent on Ab specificity [73]. Antigen–antibody (Ag-Ab) binding is converted into a colorimetric, fluorescent, optical, or electrochemical signal using labeled detection systems, such as enzymes, fluorophores, or nanoparticles (Figure 3).

### 3.1. Antibody-Based Methods

The fundamental principle of immunodiagnostic detection is the highly specific interaction between an antibody (Ab) and its corresponding antigen (Ag) or epitope (the analyte of interest) [35]. In fungal detection, these Abs target diverse fungal analytes, recognizing specific epitopes on spore walls, hyphae, extracellular polysaccharides, secreted proteins, enzymes, or mycotoxins [70,74,75]. Mycotoxin detection relies on antibodies against these small haptens, which must be conjugated to proteins or polypeptides to elicit an immune response and form an immunogen [35]. Cross-reactivity occurs when different compounds with similar chemical groups bind to the same Ab to varying degrees. Polyclonal antibodies (pAbs) consist of a mixture binding to different epitopes, while monoclonal antibodies (mAbs) are a single type specific to one epitope. While pAbs are raised in selected animal species like rodents and rabbits, mAbs are recovered from cloned cell lines. Although pAbs offer a simpler production route, they may be associated with batch-to-batch variation, whereas mAbs can provide consistent antibody characteristics and scalable production once established, despite the higher initial development effort [35]. However, analytical selectivity is not inherently determined by whether an antibody is polyclonal or monoclonal, but depends on the characteristics of the individual antibody, including its affinity and cross-reactivity. Both pAbs and mAbs are used in fungal and mycotoxin detection [35]. Recombinant Abs and its fragments have been increasingly investigated, but they remain considerably less common as standalone commercial recognition reagens [76,77]. An alternative avian Ab platform is provided by chickens, which produce immunoglobulin Y (IgY). Anti-mycotoxin IgY antibodies can be recovered non-invasively from egg yolks and have been developed against targets including ZEN and T-2 toxin [78,79]. Chicken-derived IgY has therefore attracted attention as an alternative to mammalian antibodies, particularly because Ab collection from eggs avoids repeated blood sampling from immunized animals [78]. Although commercial manufacturers rarely disclose the clonal nature and animal origin of Abs, these biological traits inherently dictate analytical thresholds through affinity and batch-to-batch variability. Consequently, the limit of detection (LOD) and limit of quantification (LOQ) are properties of the complete analytical system governed by assay format, matrix effects, and extraction efficiency rather than intrinsic limits of the Ab itself. Therefore, LOD and LOQ values cited in this review reflect the performance of the entire diagnostic method [76,77]. The cited commercial values originate primarily from comparative reviews of commercially available mycotoxin test systems, whereas the lower values represent optimized experimental immunoassays and should not be interpreted as equivalent to routine commercial kit performance [79,80,81]. From a food-safety perspective, the principal advantage of commercial Ab-based assays is therefore not necessarily an exceptionally low analytical LOD, but rather the combination of rapid analysis, relatively simple sample preparation, high throughput, portability and comparatively low cost. Many commercial immunoassays are used for screening within specified matrices and decision ranges [77,81]. Confirmation requirements depend on the intended decision and the validated method scope. Suspect screening results may require an appropriate chromatographic method, particularly for official control [77,81].

#### 3.1.1. Enzyme-Linked Immunosorbent Assays (ELISA)

**Figure 4:**
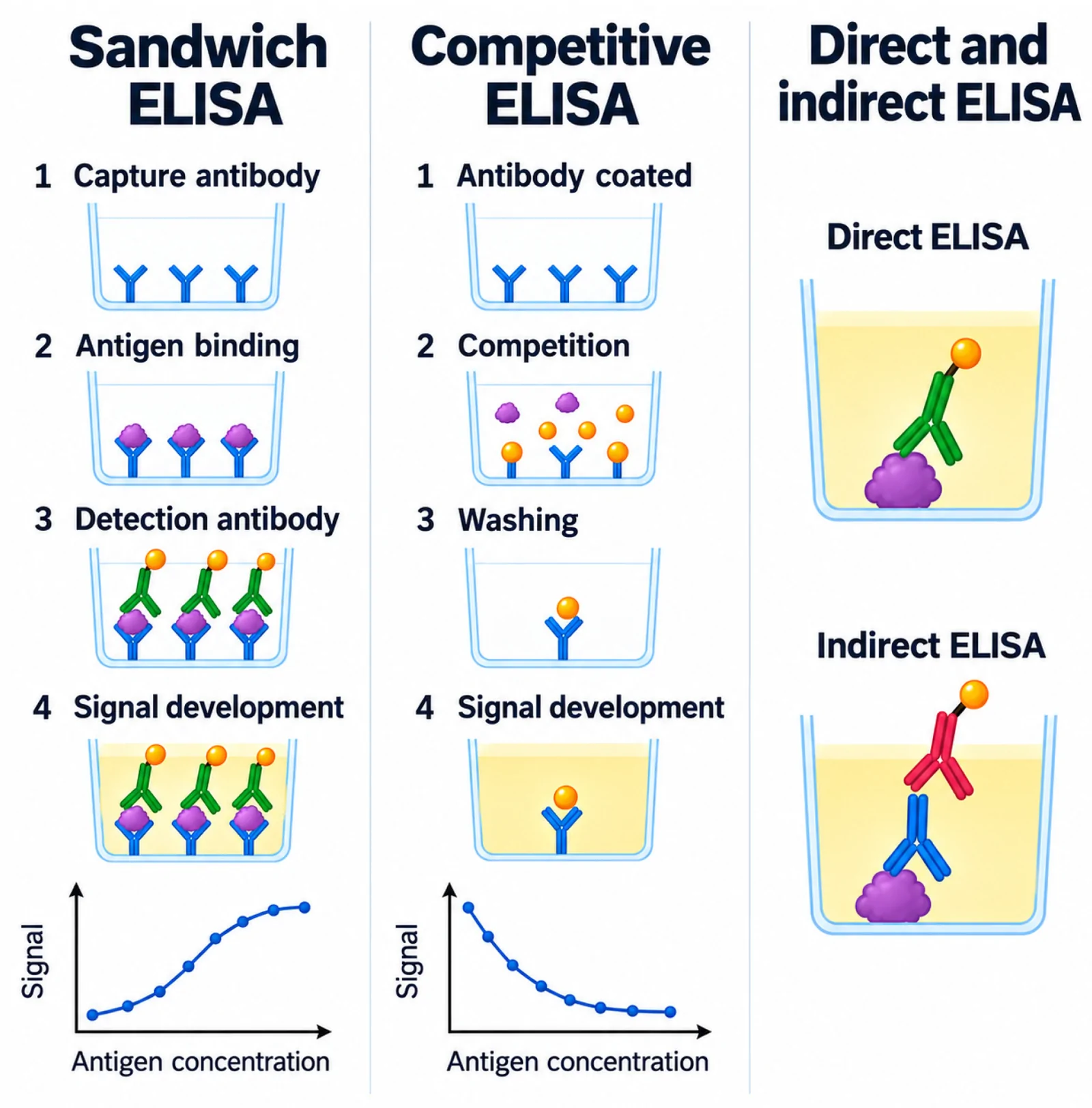
Schematic representation of ELISA formats. Sandwich recognition captures a suitable antigen between capture and detection antibodies. Competitive recognition produces decreasing signal as sample antigen concentration increases. The drawn antigen-coated surfaces illustrate direct detection with labeled primary antibody and indirect detection with secondary antibody. The measured analyte and reagent arrangement must be specified for each application. Sensitivity rankings between formats are not universal.

The enzyme-linked immunosorbent assay (ELISA) is immunological technique based on the specific binding of an enzyme-conjugated Ab to its target Ag. Following the addition of a suitable chromogenic substrate, the enzymatic reaction produces a measurable color, allowing Ag or Ab quantification [73]. There are different ELISA formats (illustrated in Figure 4), such as direct, indirect, sandwich, competitive, and multiplex assays [73]. The sandwich ELISA measures the amount of Ag that is sandwiched between two layers of antibodies and it is appropriate for intact structures and macromolecular antigens [73]. Xue et al. developed a direct sandwich ELISA combining a monoclonal capture antibody with a chicken antibody enzyme fusion [69]. The assay recognized soluble *Aspergillus* cell wall proteins and was applied to natural samples. These fungal antigen measurements must not be interpreted as aflatoxin concentrations.

Wang et al. combined the PO8 nanobody with rabbit polyclonal antibodies to detect *Aspergillus* material [82]. The nanobody recognized an approximately 45 kDa protein, whose molecular identity was not established. The analytical detection limit was 1 µg mL^−1^ of mycelial material in buffer. The reported peanut and maize threshold was 2 µg mg^−1^, expressed on a different sample basis. Reactivity extended beyond *A. flavus* and *A. parasiticus*, supporting genus recognition rather than strict species identification. Artificially contaminated and natural samples were examined, with fungal antigen and aflatoxin results not invariably agreeing.

Ansari et al. generated monoclonal antibodies against Aflmp1, a major antigenic cell wall protein of *A. flavus* [73]. Identification of the antigen improves interpretation, but food matrix performance still requires validation. Park et al. examined ELISA detection of mold contamination in agricultural commodities [74]. That broader endpoint should not be described as proof of a particular toxigenic species. Iyer and Cousin used antibodies against *Fusarium* mycelial proteins in cornmeal [75]. Their cross recognition of multiple *Fusarium* species supported screening, rather than unambiguous species assignment.

For *Alternaria*, Green et al. combined immunostaining with microscopy to examine aerosolized *A. alternata* material [63]. The method detected conidia and hyphae, including smaller fungal fragments. Its environmental application illustrates recognition of dispersed fungal material, not validation of food toxin measurement. Antigen availability may change with developmental stage and extraction conditions. Neither a positive stain nor a soluble antigen result alone demonstrates viability or toxin production [63,70].

Competitive ELISA is widely employed because small mycotoxin haptens generally cannot support conventional two-antibody sandwich recognition [76,77]. This practical constraint does not exclude specialized recognition architectures, including antibody and aptamer combinations [78]. ELISA-based immunoassays have been developed for the de-tection of several mycotoxins, including aflatoxins (AFB_1_, AFB_2_, AFG_1_, AFG_2_, and AFM_1_), OTA, DON, ZEN, fumonisin B1 (FB_1_), and fumonisin B2 (FB_2_), patulin, T-2/HT-2 toxins, and alternariol with sensitivity varying according to the food matrix and Ab specificity. Detection limits and cross reactivity depend on the complete assay and food matrix [25,64,67,68,76,77,79,80,81,83]. Performance should not be generalized from one toxin or formulation to another.

Recombinant antibodies and nanobodies offer additional recognition options, alt-hough stability and sensitivity require evaluation within each assay [84,85]. ELISA sup-ports parallel sample processing with comparatively accessible equipment [64,76,77]. Compared with instrumental analytical techniques, ELISA requires simpler sample preparation and less sophisticated equipment, making it particularly suitable for routine screening, quality control, and surveillance programs [86,87,88]. ELISA enables the rapid analysis of large numbers of samples and the efficient identification of potentially contaminated products. Its analytical performance depends on the analyte, matrix, regulatory context, and intended analytical purpose [86,87,89]. Commercial ELISA kits cover many regulated mycotoxins, but their validated matrix scope and performance differ [2,76,81].

#### 3.1.2. Lateral-Flow Immunoassays (LFIAs) and Flow-Through Immunoassays

**Figure 5:**
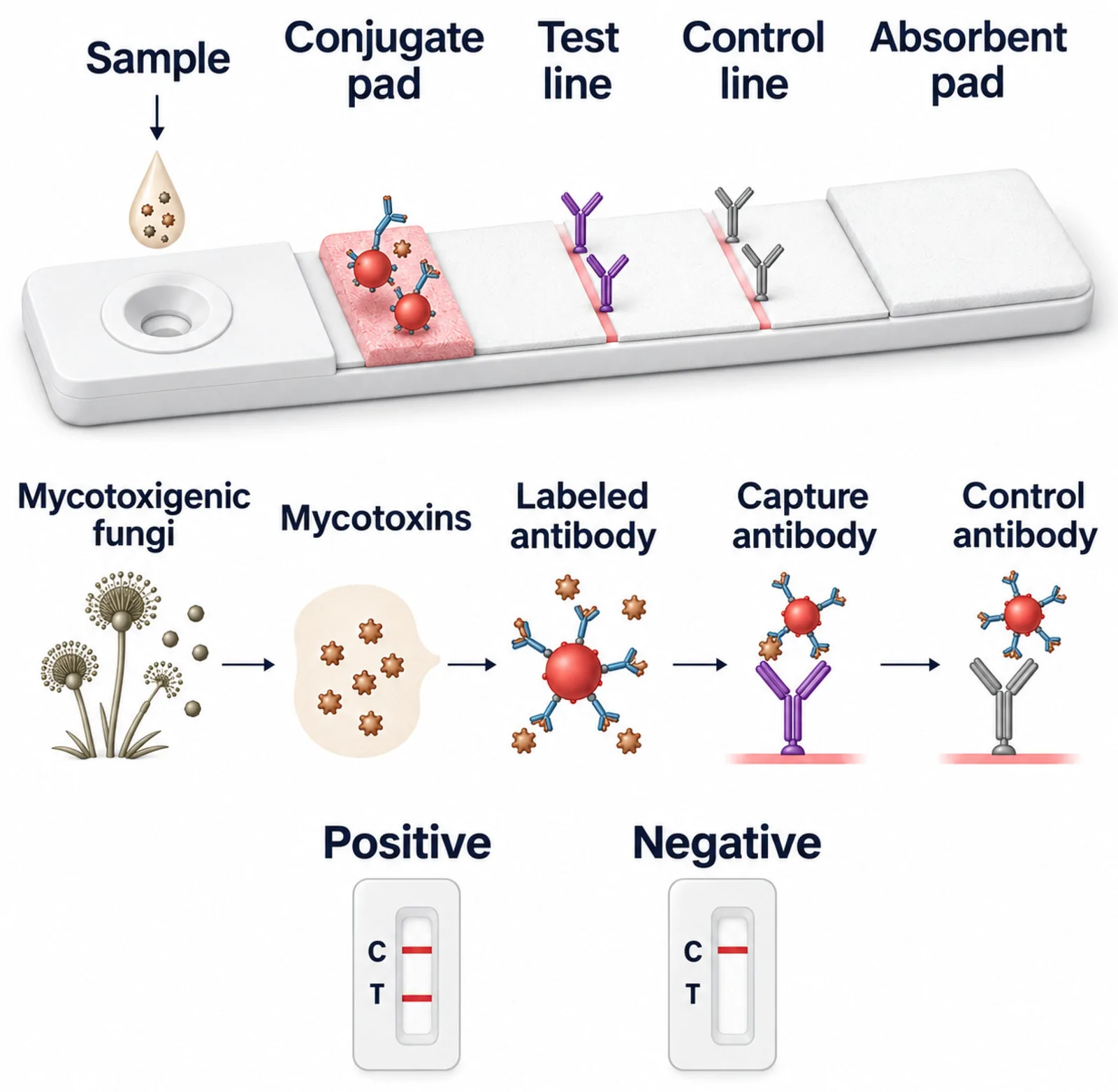
Schematic layout and signal development of a sandwich LFIA for macromolecular fungal antigens. The target presents accessible sites for capture and detection antibodies. A positive test line indicates antigen recognition within the validated assay scope. Conventional competitive mycotoxin strips use a different signal relationship, which this sandwich mechanism does not represent.

Lateral-flow immunoassay (LFIAs) is an immunodiagnostic technique based on the principles of solid-phase chromatography and specific Ag-Ab interactions (Figure 5). Specific capture reagents are immobilized in distinct zones along a porous membrane strip, such as nitrocellulose embedded into a plastic platform [90,91]. Liquid migrates by capillary action through a conjugate zone and one or more capture lines. Sandwich formats generally produce increasing test-line intensity with increasing fungal Ag, whereas competitive formats, widely used for mycotoxins, produce decreasing test-line intensity as analyte increases. Gold nanoparticles, colored latex, fluorescent particles, or other reporters provide visual or reader-assisted detection [92,93]. Wakeham et al. (2016) developed a competitive LFIA for rapid detection of *A. brassicae* conidia in air samples, with visual results obtained in approximately 6 min and a detection threshold of around 50 conidia [94].

Liang et al. investigated nanozyme assisted immunochromatographic recognition of *A. flavus* in peanut samples [95]. Their earlier colorimetric and photothermal LFIA also targeted fungal material [96]. Evaluation in one matrix does not establish elimination of interference across different foods. These studies concern fungal antigen recognition, not direct aflatoxin measurement. Commercially available LFIAs for mycotoxins in food have been validated for several mycotoxins, including AFs, and DON [91]. Adunphatcharaphon et al. (2024) designed a portable, microarray-based lateral-flow system enabling the concurrent, on-site quantification of AFB_1_, T-2 toxin, ZEN, DON, and FB_1_ in rice [97]. Xing et al. investigated a time resolved fluorescent LFIA for AFB_1_ screening in peanuts and maize [98]. Immunochromatographic assays are increasingly used as rapid, portable tools for visual yes/no detection and semi-quantification of mycotoxins, with multiplex platforms enabling simultaneous detection of multiple toxins [2].

Visual LFIAs commonly provide qualitative or semiquantitative interpretation, whereas calibrated readers can support quantitative results [86,99]. Adunphatcharaphon et al. reported quantitative analysis of five mycotoxins in rice using a portable fluorescence reader [99]. Their extraction to readout workflow required 17 min, excluding initial sampling and milling. Detection limits ranged from 0.56 to 1.89 µg kg^−1^ across the five targets. These figures describe that assay and rice matrix, not LFIAs generally. Results outside validated ranges, or requiring official confirmation, should follow the relevant reference procedure [98].

Flow-through immunoassays direct liquid perpendicularly through a capture membrane. Convective transport can shorten incubation and washing, and discrete capture spots support multiplexing. Beloglazova et al. 2017 used a flow-through format to screen ZEN, DON, AFB_1_, and OTA in cereal-derived feed ingredients [100].

#### 3.1.3. Optical Immunodiagnostic Platforms

Fluorescent immunoassays (FIA) and chemiluminescent immunoassays (CLIA) are optical immunodiagnostic platforms that employ fluorescent labels and chemiluminescent reactions, respectively, to generate measurable light signals following Ag-Ab recognition [101,102]. Compared with conventional colorimetric assays, FIA and CLIA can provide high sensitivity, broad dynamic ranges, and improved quantitative performance in appropriately designed assays, although these analytical characteristics depend on the specific assay, analyte, and sample matrix. Their performance may also be affected by Ab specificity, cross-reactivity, and matrix-related interferences, particularly in complex food matrices [63,79,103]. In the field of food safety, these characteristics make them suitable for detecting major mycotoxins [76,78,104]. Recent developments have focused on integrating these methods with portable fluorescence and chemiluminescence readers, smartphone-assisted imaging systems, and microfluidic platforms, facilitating rapid and quantitative analysis beyond conventional laboratory settings [105,106]. Quantum dot-based fluorescent microsphere immunoassays have already demonstrated highly sensitive simultaneous detection of AFB_1_, DON, and ZEN in cereal samples. The result concerns that assay architecture rather than a universal advantage of optical labels depends on the specific assay and analyte [107].

#### 3.1.4. Immunosensor Platforms

Immunosensors are affinity based solid state biosensors in which the target Ag is detected through Ab binding, generating a measurable signal by the transducer [108]. Although immunosensors operate similarly to immunoassays, they differ in that Ag-Ab recognition and signal detection occur on the same platform [109]. They are classified according to their transduction mode as electrochemical, optical, and piezoelectric devices [108,110], with each type employing specific analytical approaches and different labelling strategies to enhance detection capabilities [111]. Sample consumption and instrument requirements differ between immunosensor designs [108,111,112]. Low analytical detection limits do not themselves demonstrate stable performance in naturally contaminated foods. In food safety applications, these immunosensors have been predominantly developed for detection of mycotoxins, such as AFB_1_, OTA, FBs, DON, ZEN, T-2 toxin, and patulin in cereals, nuts, feed, and other agricultural commodities [70,72,113]. Surface plasmon resonance-based immunosensors have also enabled simultaneous detection of multiple mycotoxins, including AFB_1_, OTA, ZEN, and DON, in cereal matrices using competitive immunoassay strategies [114,115]. Bonel et al. developed competitive electrochemical immunosensors for the detection of OTA based on disposable screen-printed electrodes. The sensor design involved OTA–bovine serum albumin (OTA–BSA) conjugates and gold nanoparticle-assisted nanostructuring of the electrode surface, enabling sensitive OTA determination through differential pulse voltammetry. The developed immunosensors were successfully evaluated using wheat samples, demonstrating the applicability of electrochemical platforms for mycotoxin monitoring in cereals [116]. An improved OTA immunosensor was later developed using a biotinylated mAbs and colloidal gold nanostructuring, providing enhanced analytical performance and successful validation in wheat samples [117]. Electrochemical impedance spectroscopy (EIS) has also been applied for label-free detection of AFB_1_, one of the most important foodborne mycotoxins. An impedance-based immunosensor using a gold electrode modified with self-assembled layers and an AFB_1_–BSA conjugate as the recognition interface was developed. Specific Ab binding caused measurable changes in interfacial impedance, enabling sensitive detection of AFB_1_ in food-related samples [118].

Lee et al. developed a microgap immunosensor recognizing extracellular proteins of *A. niger* [119]. This fungal recognition example should not be described as direct mycotoxin detection. Its reported application also does not establish validation across naturally contaminated food matrices. Antigen selection and testing against closely related species determine the useful taxonomic specificity. Comparison with culture is needed when viability, rather than antigen presence, is the analytical question [120].

The main challenges encountered by different types of immunosensors include sensor stability, matrix interference, surface regeneration, and the transfer from laboratory-based systems to reliable field instruments [72,108,121,122]. Microfluidic and lab-on-a-chip platforms have therefore emerged as promising approaches for addressing some of these challenges through compact, efficient, and portable immunosensing systems [113].

## 4. Aptamer-Based Recognition and Aptasensors

Aptamers have emerged as promising biorecognition elements for the development of rapid, sensitive, and selective biosensors, primarily for the detection of mycotoxins, but they can also be used for the detection of mycotoxin-producing fungi [123,124]. They are synthetic single-stranded DNA (ssDNA) or ribonucleic acid (RNA) oligonucleotides with high specificity and affinity toward their target [125] that may include small molecules, proteins, toxins, components of the cell surface, and whole cells [126]. Aptamers of ssDNA that are specific for a target are selected from combinatorial libraries of synthetic nucleic acids through exponential enrichment via an iterative process of target binding, recovery, and amplification, known as Systematic Evolution of Ligands by EXponential enrichment (SELEX) [127,128]. Chemical synthesis facilitates aptamer modification and reproducible manufacture [72,113]. Stability and binding still depend on sequence, folding conditions, matrix composition, and storage. These properties require experimental evaluation rather than assumption. During the past decade, aptamer technology has been applied to the determination of AFs, OTA, FUM, ZEN, and T-2 toxin [123]. Recent advances include the development of aptasensors based on different signal-transduction strategies, particularly electrochemical, optical, and photoelectrochemical approaches [123,129]. The incorporation of advanced nanomaterials, including gold nanoparticles, materials based on graphene, MXenes, and metal-organic frameworks, has enhanced signal amplification and analytical sensitivity in mycotoxin detection using aptasensors [130,131,132]. On the other hand, the direct detection of mycotoxigenic fungi using aptamers remains comparatively limited [133,134]. SELEX-based approaches offer great potential for selecting aptamers that recognize fungal spores, cell-wall components, and fungal-specific proteins. Seo et al. selected spore binding aptamers that recognized several *Aspergillus* species [135]. Such recognition does not prove that the detected material is viable or producing toxins. Low et al. instead selected aptamers against β-glucans [136]. Recognition of that shared wall component is not equivalent to species identification [135,136]. Multiplex aptasensors have been shown to simultaneously detect multiple mycotoxins by coupling distinct recognition probes with optical or electrochemical signal outputs [134,137,138].

Ellena et al. subsequently selected aptamers against *A. niger* conidia using counterselection with the closely related *A. tubingensis* [125]. Binding was also assessed against *A. nidulans*. This design addresses a specificity problem that positive target binding alone cannot resolve. The work involved cultured conidia, rather than validation in naturally contaminated foods. Binding affinity should not be reported as an assay detection limit. Neither the earlier spore study nor this selection establishes direct toxin measurement [121,125].

Zhang et al. (2026) reported a dual-mode photoelectrochemical-colorimetric aptasensor for highly sensitive detection of AFB_1_ using a BiVO_4_/Ni_3_(HHTP)_2_ heterojunction and PdAuCu nanoenzyme amplification [139].

A summary Table showing analytical comparison of representative detection platforms, including operational characteristics and implementation status (Table 1).

## 5. Imaging-Based Detection and Artificial Intelligence

Spectral imaging combines spatial information with wavelength dependent measurements of a sample [127]. Multispectral imaging (MSI) uses selected spectral bands, whereas hyperspectral imaging (HSI) acquires more densely resolved spectra. Exact band counts and widths depend on the instrument rather than a universal numerical boundary. Existing maize studies illustrate screening associated with AFB1 contamination [65,128,129]. Bartolić et al. combined fluorescence spectroscopy and MSI for preliminary fingerprinting of contaminated maize seeds [65]. Such fingerprints require calibration and must not be interpreted as direct fungal identification. Red, green, and blue (RGB) images provide additional color and texture information but no inherent toxin specificity.

Recent primary studies provide more informative tests of the connection between images and food contamination. Borràs-Vallverdú et al. examined 98 naturally contaminated maize samples without visible fungal infection [130]. NIR-HSI models were compared with chemically measured DON and fumonisins. Reported cross validation sensitivity was 0.778 for DON and 0.607 for combined fumonisins. Corresponding specificities were 1.000 and 0.938 at the thresholds used in that study. Those results support screening research, while the missed positives caution against assuming regulatory readiness. Multiple scans came from each sample, making validation at the independent sample level especially important. Study thresholds should not be substituted for the currently applicable legal limits.

Nie et al. combined HSI with deep learning to predict fungal contamination indicators in millet [131]. Samples originated from 12 production areas, but contamination measurements followed controlled storage enrichment for 30 days. HPLC measurements supplied reference values for ergosterol and DON. Ergosterol represented fungal biomass related information, whereas DON was a toxin endpoint. These predictions therefore answer different analytical questions. The enriched storage conditions also limit extrapolation to untreated commercial lots. Wider geographical sampling does not, by itself, establish external validation of contamination predictions.

Dhakal et al. used hyperspectral images and machine learning to distinguish damaged wheat kernels [132]. That classification endpoint differs from chemical determination of a particular mycotoxin. Javidan et al. investigated RGB and hyperspectral signatures of experimentally induced tomato fungal diseases [133]. Their work included *Alternaria* and *Fusarium*, but disease recognition did not establish toxin concentrations. Such studies are useful methodological context rather than evidence of validated mycotoxin measurement. Their applicability to harvested food should be tested using corresponding reference chemistry.

Portable instruments offer a different route toward practical screening. Smeesters et al. developed a handheld fluorescence spectrometer and examined naturally contaminated maize kernels [134]. Benchmarking against a research instrument supported feasibility, but the study did not establish universal lot acceptance performance. Commercial imaging hardware was used in the maize study, including a Resonon Pika NIR camera [130]. The millet study employed HySpex imaging equipment [131]. Availability of those instruments does not amount to commercial validation of the authors’ contamination models.

Smartphone imaging of an assay must be distinguished from direct photography of food. A phone may quantify a calibrated strip signal, while a photograph of grain supplies different information. The rice multiplex LFIA used a purpose-built portable fluorescence reader rather than ordinary phone photography [86]. Smartphone compatible optical assays remain attractive, provided illumination and image acquisition are controlled [135,136]. Phone model changes and automatic exposure settings can alter quantitative interpretation. Validation must therefore cover the complete reader and assay combination, not the camera alone.

AI evaluation should separate model development from genuinely independent testing. Images or repeated scans from one kernel or sample should remain within the same data partition. Otherwise, apparent performance may partly reflect repeated observation of the same material. External lots and harvest years provide a stronger test of transferability. Cultivar differences, moisture, illumination, instrument response, storage history, and heterogeneous contamination can alter the measured features [130,131,137]. Performance near the intended decision threshold deserves particular attention. Imaging can prioritize samples for chemical analysis without being presented as a universal replacement for it.

## 6. Sample Preparation

Sample preparation is a critical determinant of the downstream analytical performance of fungal and mycotoxin assays. It dictates the specific fraction of the target entering the analytical workflow, directly influencing recovery rates, matrix effects, repeatability, and the LOD [142,143]. Conidia and hyphal material may be distributed unevenly within food matrices [144,145]. The fungal structures present depend on the species and developmental conditions. Representative sampling must precede extraction, because analytical sensitivity cannot compensate for an unrepresentative test portion [146,147]. Consequently, the extraction strategy must be tailored to the distinct analytical endpoint. While molecular assays require the efficient release and purification of nucleic acids, immunoassays demand the preservation of accessible antigenic determinants [148,149,150]. Conversely, mycotoxin analysis relies on the validated extraction of low-molecular-weight metabolites from these target complexes [12,149]. Therefore, sample preparation must be viewed as an integrated component of the diagnostic methodology rather than an isolated, preliminary step [142].

### 6.1. Fungal Structures and Lysis Requirements

The fungal cell wall is a dynamic and structurally heterogeneous barrier composed primarily of chitin and branched glucans, together with mannans, galactomannans, proteins, melanins, hydrophobins and other polymers [151,152]. The relative abundance and organization of these components vary among species, developmental stages and environmental conditions [145]. Consequently, conidia of *A. flavus*, macroconidia and chlamydospores of *F. verticillioides*, and conidia of *A. alternata* may exhibit different mechanical resistance, surface hydrophobicity, enzyme accessibility and chemical reactivity [153,154,155,156].

Fungal morphology and cell-wall characteristics can substantially influence lysis efficiency [157,158,159]. Dormant, melanized or heavily pigmented spores may be more resistant to disruption than actively growing hyphae [160]. DNA recovery can depend on spore maturity, pigmentation, cell-wall composition and extracellular material [157,158,159]. Environmental fungal samples may contain mixtures of mycelium, yeast-like cells, sexual and asexual spores, and DNA recovery can therefore differ among these forms [157,158]. Hyphal material introduces additional challenges. Hyphae may remain attached to plant fibers, become embedded in extracellular matrices or distribute unevenly during homogenization [157]. Hydrophobic conidia may disperse poorly in aqueous buffers and form aggregates, which can reduce the number of target structures entering the analytical aliquot [161]. Therefore, method validation should use the developmental forms and matrices expected in the intended application. Cultured vegetative mycelium should not be considered a universal surrogate for field conidia, chlamydospores or sclerotia material [162,163].

Comparative studies of fungal cell disruption and nucleic acid extraction have demonstrated substantial species- and morphology-dependent differences in extraction efficiency. Enzymatic lysis provided high DNA recovery from *Candida albicans*, but was considerably less effective for *Aspergillus fumigatus* conidia and hyphae, whereas bead-mediated mechanical disruption yielded higher DNA recovery from *A. fumigatus* [158]. Similarly, it has been demonstrated that no single combination of physical, chemical, and enzymatic treatments was optimal across the 16 fungal species examined [164].

These DNA extraction results cannot establish preservation of antibody binding sites. Antigen extraction must be assessed directly, because disruption can improve release while altering recognition [151,152]. Pogner et al. found species dependent differences between fungal antigen measurements and other airborne fungal measurements [70,145]. Their study concerned air samples, not validation in foods. Antigen detection persisted after loss of culturability in the investigated material. This illustrates why ELISA signals cannot be treated as viable counts.

### 6.2. Matrix Complexity and Inhibition

Food, plant, and environmental samples represent chemically complex matrices that can interfere with fungal Ag recovery and immunodetection. Cereals and seeds contain abundant starch, proteins, lipids, and structural components, whereas fruits contain pectin, pigments, phenolics, waxes, and lignocellulosic material [165,166,167,168]. Evidence from food allergen extraction also shows how matrix composition can affect protein recovery [169,170]. Such findings inform fungal antigen method development but do not replace direct validation with fungal targets.

Matrix components can influence fungal detection at different stages of the analytical workflow. Polysaccharides and polyphenolic compounds may co-extract or co-precipitate with nucleic acids reducing DNA purity and affecting downstream amplification, while oxidized phenolics can interact irreversibly with DNA and proteins [171]. Metal ions can inhibit DNA amplification, with effects depending on their identity and concentration [172]. In lateral-flow and microfluidic platforms, variations in sample viscosity and the presence of particulate or lipid-rich material can alter capillary flow and affect signal development [173]. In addition, naturally occurring pigments and other optically active components of matrix may interfere with absorbance- or fluorescence-based readouts [174]. Matrix-associated inhibition can be reduced by adapting extraction procedures to the composition of the sample. Modified cetyltrimethylammonium bromide (CTAB) protocols incorporating additives such as polyvinylpyrrolidone (PVP) or insoluble polyvinylpolypyrrolidone (PVPP) and reducing agents have been used to improve nucleic acid recovery and purity from plant tissues rich in polysaccharides and polyphenols [175,176]. Matrix effects are also relevant to immunoassays, as co-extracted matrix constituents may affect Ag recovery, Ab binding, and assay signal. Therefore, fungal detection methods should be evaluated using matrix-matched or matrix-spiked samples rather than purified targets alone [177,178].

### 6.3. Representative Sampling and Homogenization

Representative sampling is a critical first step in fungal and mycotoxin analysis because contamination can be highly heterogeneous within food and plant materials. In particular, aflatoxin contamination in cereals may be unevenly distributed, making sampling and subsampling major sources of analytical variability [49]. Adequate sample mass, multiple increments, mixing, and thorough homogenization are therefore essential for obtaining reliable results [179,180]. Incremental samples should represent the relevant lot before they are combined into an aggregate sample [181]. Laboratory homogenization then precedes selection of the analytical test portion. Repeated measurements from one extract assess a different source of variation than independent subsampling. Unevenly contaminated kernels can dominate aflatoxin uncertainty even when instrumental repeatability is acceptable [146,147]. Sampling plans should follow the applicable commodity and analytical purpose, rather than a universal sample mass.

Homogenization and cellular lysis should be considered distinct operations. Bulk homogenization reduces spatial heterogeneity, but cannot correct an initially unrepresentative collection [146,147]. Subsequent lysis releases intracellular targets; intact spore assays may instead require preservation of their recognition surfaces [70,120,121]. Recent work using a micro-homogenizer for citrus tissues demonstrated that rapid mechanical disruption can generate nucleic acids suitable for quantitative real-time PCR, although dilution or simple cleanup may be required to reduce residual inhibitors [182].

### 6.4. Chemical and Enzymatic Lysis

Chemical lysis typically combines detergents such as sodium dodecyl sulfate (SDS) or CTAB with proteinase K and, when required, PVP or reducing agents to disrupt fungal cells and remove polysaccharides and phenolic inhibitors [183,184]. Modified CTAB/SDS protocols can provide high-quality DNA from plant- and fungus-rich matrices, although organic extraction increases processing time and handling requirements [183,184,185].

Enzymatic treatments can disrupt fungal walls under comparatively mild conditions [142,168]. Their effects on the intended antigen must nevertheless be measured separately from DNA recovery. Given the variability in fungal cell-wall composition, enzymatic treatment is generally more appropriately considered as a complementary lysis step rather than a universal strategy for fungal cell disruption [185]. Differences in susceptibility to cell-wall-degrading enzymes have been demonstrated for filamentous fungi, including *Fusarium* spp. [185,186].

### 6.5. Mechanical Disruption and Bead Beating

This approach is particularly effective for fungal structures resistant to chemical or enzymatic lysis. Bead beating provides a standardized and relatively reproducible method in a closed vessel, with efficiency depending on bead characteristics, processing conditions, fungal species, and sample matrix [169,170]. It can substantially improve fungal DNA recovery, including from *Aspergillus* spp., although disruption conditions may affect the relative recovery of different fungal species [169,170,171]. Rapid platforms such as FastPrep enable standardized and parallel processing of fungal and plant samples [173], while comparative studies indicate that different platforms can produce different fungal DNA yields [167,174].

The process should be optimized rather than maximized [172]. Insufficient disruption may reduce target recovery, whereas excessive processing can cause DNA shearing and compromise nucleic-acid integrity [167]. Therefore, platform and extraction conditions should be selected according to the sample matrix and downstream analytical endpoint and evaluated using matrix-matched samples [175].

### 6.6. Crude Extracts and Mycotoxin Cleanup

Crude-extract strategies can be advantageous for qualitative screening when the target concentration is sufficiently high and the downstream assay tolerates residual inhibitors. Dilution is the simplest form of cleanup because it reduces inhibitor concentration, although it simultaneously decreases target concentration. It is useful only when the remaining target concentration exceeds the effective detection limit [187,188].

More extensive purification is required when fungal burden is low, quantitative accuracy is important, matrix composition varies considerably, or false-negative results have serious consequences. For molecular assays requiring high analytical sensitivity and reliable quantification, such as PCR-based detection of mycotoxigenic fungi, DNA integrity and purity may be more important than achieving the shortest possible extraction time [189,190]. For immunodiagnostic platforms, insufficient cleanup may cause nonspecific interactions and signal suppression or enhancement, reducing assay accuracy. Therefore, crude extracts should be evaluated for matrix effects, dilution linearity, and extraction recovery, particularly when quantitative results are required or when different food matrices are compared [26,191].

Mycotoxin cleanup should be chosen according to analyte chemistry and the food matrix, rather than detection format alone [192]. QuEChERS combines solvent partitioning with salt assisted extraction and, frequently, dispersive solid phase extraction (SPE). Its name denotes quick, easy, cheap, effective, rugged, and safe preparation. Broad analyte coverage is useful, although polar toxins may partition differently and sorbents can retain intended analytes. Facorro et al. combined QuEChERS with SPE for multiple mycotoxins in mixed feed rations [193]. Their method illustrates a matrix specific solution, not equal recovery of every toxin.

Immunoaffinity columns (IACs) selectively retain toxins recognized by their immobilized antibodies [149,194]. They can reduce interference, but capacity and solvent compatibility constrain their use. Unrecognized modified toxins may be lost during cleanup. Conventional SPE offers different retention chemistries without requiring an antibody, although coextractives and analyte losses still require testing [143,194]. Selection of the sorbent therefore changes both cleanliness and analytical coverage. A cleaner extract is not necessarily a more representative toxin profile.

Dilute and shoot workflows avoid selective cleanup by diluting the extract before instrumental injection. They preserve broad coverage but also introduce residual matrix components. Sulyok et al. validated this approach for more than 500 microbial metabolites across seven food matrices [195]. Extraction recovery and apparent recovery differed because ion suppression or enhancement varied with matrix. Their results show why matrix effects require examination across independent samples. Dilution also raises the effective detection limit in the original food unless compensated by validated instrumental sensitivity.

Cereal extracts can contain starch and proteins, whereas nut extracts introduce substantial lipid interference [192,195]. Acidic or pigmented fruit matrices create different extraction and optical problems. Compound feed adds variable ingredients and processing histories, complicating transfer between formulations [193]. Puntscher et al. evaluated free and modified *Alternaria* toxins in tomato sauce, sunflower oil, and wheat flour [196]. Their results should be interpreted for those matrices rather than all foods. Antigen assays require separate attention because solvents suitable for toxin extraction may impair protein recognition [169,197]. Harsh disruption can release more antigen while also changing its accessible epitopes. Matrix blanks and recovery experiments should accompany comparisons between mild extraction and aggressive lysis.

### 6.7. Integrated Sample Preparation and Detection

Sample preparation and detection should be considered as a single analytical workflow, because incomplete homogenization or lysis can reduce target recovery and lead to false-negative or underestimated results. This is particularly important for heterogeneous food matrices, where fungal contamination may be unevenly distributed [198,199].

Quality control should therefore cover the entire procedure, from homogenization to detection. Process controls introduced before extraction, extraction blanks, matrix-matched negative controls, and internal amplification or detection controls can help distinguish target loss during sample preparation from assay failure. Replicate analysis and comparison with an established reference method can further assess method robustness [200,201]. ISO 16140-2 addresses validation of alternative microbiological methods, not chemical determination of mycotoxins [202]. Official mycotoxin control requires the relevant sampling and analytical performance criteria, including Regulation (EU) 2023/2782 [181].

Analytical performance should be evaluated using purified targets as well as spiked and naturally contaminated samples. Matrix-spike experiments provide information on recovery and matrix effects, whereas naturally contaminated samples better reflect the heterogeneity and target distribution encountered in real food matrices [12,80,194].

Fortification after extraction cannot establish recovery during extraction. Naturally contaminated samples also test analyte distribution and binding that a purified standard may not reproduce [184]. Validation should examine reagent lots and storage stability, alongside performance near the intended decision concentration. Testing by different analysts or laboratories provides stronger evidence of reproducibility than repeated measurements on one device [67,181,202]. Experimental demonstrations should remain identified as such until their intended matrices and decisions are supported.

**Figure 6:**
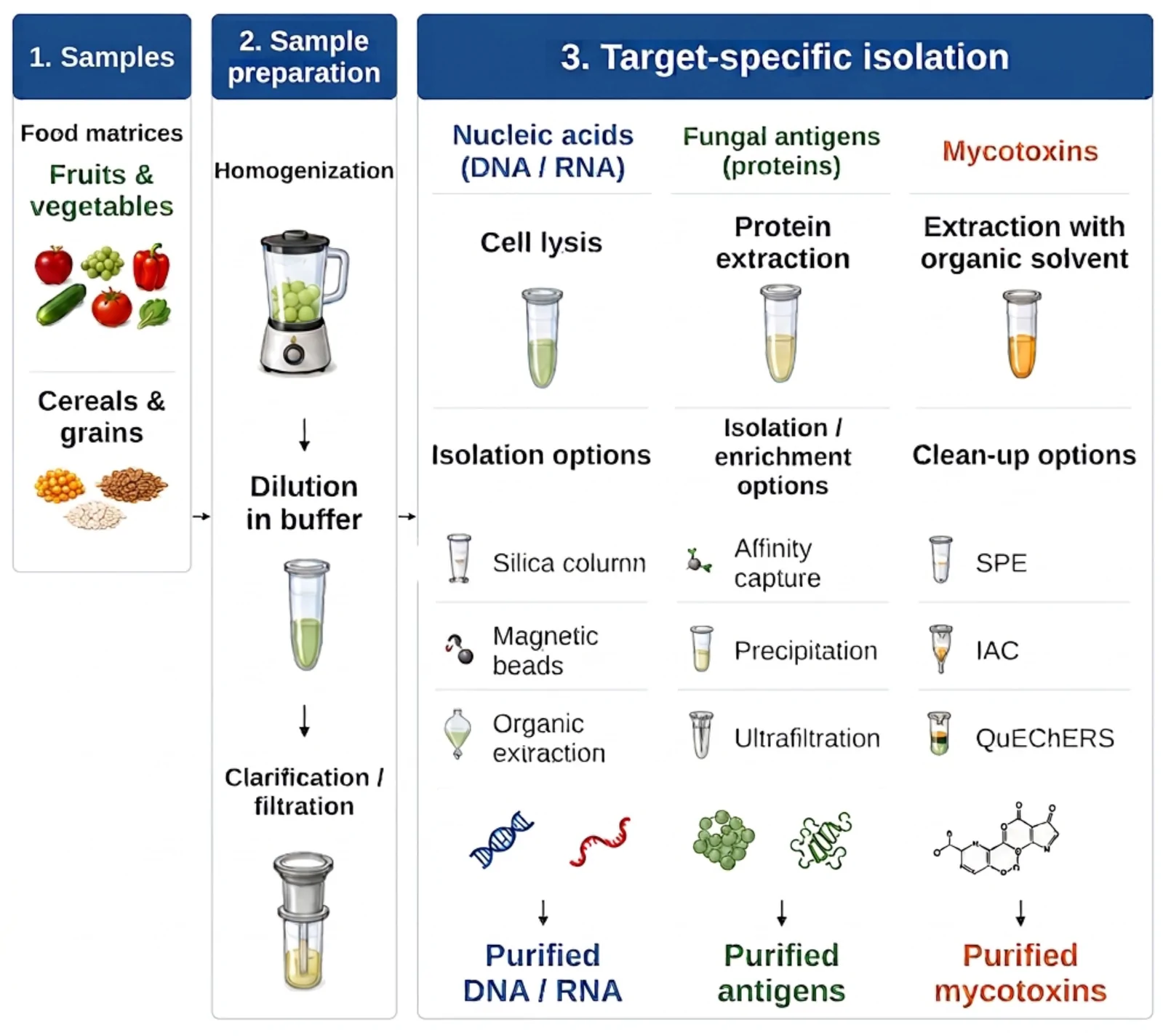
Workflow for target-specific isolation of nucleic acids, fungal antigens, and mycotoxins from food matrices. SPE, Solid-Phase extraction; IAC, Immunoaffinity Chromatography; QuEChERS, Quick, Easy, Cheap, Effective, Rugged, and Safe method.

Workflow for target-specific isolation of nucleic acids, fungal antigens, and mycotoxins from food matrices is presented in Figure 6.

## 7. Future Perspectives

The future development of biosensing platforms for toxigenic fungi and mycotoxins is increasingly focused on the transition from laboratory-based analysis toward rapid, portable, and field-deployable diagnostic systems. Although chromatographic methods remain essential for confirmatory mycotoxin analysis, optical and electrochemical biosensors offer attractive opportunities for rapid screening and decentralized monitoring across the agricultural and food production chains [72].

A major research direction is the development of multiplex and multimodal platforms capable of simultaneously detecting fungal biomarkers and their associated mycotoxins. Such systems could measure fungal markers and toxins through separate recognition channels. A fungal marker alone cannot establish the concentration of its associated toxin. This could help overcome the limitations of conventional workflows, in which fungal identification and mycotoxin determination are performed separately. Optical approaches, including fluorescence, SPR, surface enhanced Raman spectroscopy (SERS), and colorimetric sensing, as well as electrochemical platforms based on differential pulse voltammetry (DPV), square wave voltammetry (SWV), and Electrochemical Impedance Spectroscopy (EIS), are particularly promising for multiplex analysis [203,204,205].

The integration of nanomaterials, including gold nanoparticles, graphene derivatives, carbon nanotubes, quantum dots, MXenes, and metal–organic frameworks, is expected to further improve signal amplification, sensitivity, and multiplexing capabilities. In parallel, advances in aptamer selection, SELEX, and bioinformatics-assisted optimization may facilitate the development of recognition elements capable of targeting both mycotoxins and intact fungal cells [124,133,134].

Another important trend is the miniaturization and digital integration of biosensors through screen-printed electrodes, microfluidics, portable optical readers, and smartphone-compatible devices. Coupling these platforms with AI-assisted signal processing could reduce operator-dependent interpretation and enable decentralized, on-site screening in crops, storage facilities, and food processing environments [206,207,208].

Despite these advances, a major challenge remains the translation of promising proof-of-concept results obtained under optimized laboratory conditions into reliable real-world applications. Many biosensor studies demonstrate high analytical sensitivity and selectivity using controlled samples, whereas routine food-safety applications require validation across relevant and diverse food matrices, naturally contaminated samples, laboratories, and production batches. Sensor stability, reproducibility, matrix effects, cross-reactivity in multiplex assays, and standardized performance criteria must also be systematically addressed. Comparison with established reference methods and harmonization with regulatory requirements will be essential for demonstrating analytical reliability and practical applicability. Addressing these limitations will be critical for translating advanced biosensors from laboratory proof-of-concept systems into robust tools for early contamination screening and preventive food safety management.

## 8. Conclusions

The rapid and reliable detection of mycotoxigenic fungi and their toxins is essential for effective food safety monitoring and timely risk management across the food and feed chain. Culture establishes recovery of viable fungi under selected conditions, while molecular methods detect specified DNA targets. Biosynthetic genes indicate potential toxin production, not measured contamination. Direct toxin analysis remains a separate endpoint, and antigen recognition alone does not establish viability. Affinity-based platforms have expanded the possibilities for rapid screening offering advantages in terms of speed, simplicity, portability, and potential for on-site analysis. However, sample preparation remains a critical bottleneck, as fungal cell wall’s structure and complex food matrices can significantly affect analytical performance and lead to matrix interference or false results. Future developments should connect representative sampling with extraction and validated detection. Naturally contaminated foods and independent testing remain necessary before prototypes support routine decisions. Appropriate controls and portable readouts may then improve practical monitoring. Such advances could facilitate sensitive and cost-effective point-of-need detection and strengthen early surveillance and control of fungal contamination and mycotoxins, ultimately supporting safer food and feed systems.

## Figures and Tables

**Table 1:** (**A**). Analytical comparison of representative detection platforms. (**B**). Operational characteristics and implementation status of the same platforms.

(**A**)			
**Platform and Endpoint**	**Sensitivity and Specificity**		**Matrix Tolerance and Preparation**
Culture: culturable fungal propagules [20,48,49]	No universal analytical LOD. Recovery depends on test portion and growth conditions. Enumeration alone does not identify species.		Homogenization, dilution, plating, and incubation. Injured or poorly growing fungi may be missed; spreading colonies can obscure others.
PCR: fungal DNA or specified biosynthetic genes [36,40,53,55,71]	Assay specific LOD; not a viability or toxin measurement. Primers require testing against related taxa. A common numeric LOD is not reported.		DNA extraction and inhibition controls are required. Matrix tolerance depends on purification and amplification chemistry.
ELISA: fungal antigens or mycotoxins [67,75,82]	Example: PO8 *Aspergillus* assay, 1 µg mL^−1^ mycelial material in buffer. Peanut and maize threshold: 2 µg mg^−1^. Genus cross recognition limits species assignment.		Antigen preserving extraction or validated toxin extraction. Dilution and matrix controls are necessary; the cited fungal assay included natural samples.
LFIA: fungal antigens or mycotoxins [86,87,140,141]	Example: five toxin rice assay, LODs 0.56 to 1.89 µg kg^−1^. Those values do not apply to fungal strips. Specificity depends on the capture reagents.		Rice example uses extraction and dilution before reader analysis. Lipids, particles, and viscosity can affect flow; transfer to other foods needs validation.
Immunosensors: antibody recognized antigen or toxin [105,106,107,108,109,110]	Individual assays report target dependent sensitivity. No common LOD is reported here. Fungal protein recognition does not demonstrate toxigenicity.		Extraction and sensor surface fouling require control. Wheat and cereal examples cannot establish performance for every food.
Aptasensors: selected molecular target or fungal surface [78,121,124,125]	No common LOD. Aptamer binding affinity is not an assay LOD. Related *Aspergillus* species must be tested; viability is not established by binding.		Selection buffer and food extract differ. Folding, nonspecific adsorption, and extraction recovery require matrix specific testing.
(**B**)			
**Platform**	**Time and Cost Basis**	**Portability**	**Validation and Commercial Availability**
Culture [20,48,49]	FDA workflow: count after 5 days, with longer incubation when needed. Low basic equipment expenditure; substantial incubation and analyst time.	Requires incubation and microbiological handling; not an immediate field result.	Established food enumeration workflows and commercial media. Species identification and toxin production require additional work.
PCR [36,40,55,71]	Extraction plus amplification generally requires hours; exact total varies. Instrument expenditure exceeds basic culture; reagent costs depend on format.	Portable thermocyclers do not remove extraction requirements.	Published food assays exist. Availability of instruments or reagents is not validation of every fungal target and matrix.
ELISA [67,75]	Multiple incubation and wash steps; complete fungal workflow time not reported here. Plate reader expenditure and recurring reagent costs.	Laboratory plate assays are less portable than strips.	Commercial mycotoxin kits have specified scope. The cited fungal recognition systems are research assays, not demonstrated marketed kits.
LFIA [86,87,140,141]	Rice example: 17 min from extraction to readout, excluding sampling and milling. Strip consumables; quantitative formats add reader expenditure.	Visual strips are portable. Quantitative systems may require a dedicated reader.	Commercial toxin strips and published validation studies coexist with experimental fungal strips. Cited rice results include comparison with LC-MS/MS.
Immunosensors [105,106,107,108,109,110]	Assay dependent; a comparable complete turnaround time is not reported. Transducer and fabrication costs vary considerably.	Portable electrochemical readout is possible; some optical platforms remain laboratory based.	Food matrix demonstrations exist. Broad natural sample and interlaboratory validation cannot be inferred from prototype performance.
Aptasensors [78,121,124,125]	Preparation and signal development are assay dependent; complete time not reported uniformly. Synthesis and reader costs are design dependent.	Portable array readers have been demonstrated.	Mostly experimental examples in the cited literature. Fungal selection studies do not establish validated commercial food tests.

## Data Availability

No new data were created or analyzed in this study. Data sharing is not applicable to this article.

## References

[1] Li P., Tedersoo L., Crowther T.W., Wang B., Shi Y., Kuang L., Li T., Wu M., Liu M., Luan L. (2023). Global Diversity and Biogeography of Potential Phytopathogenic Fungi in a Changing World. Nat. Commun..

[2] Anfossi L., Giovannoli C., Baggiani C. (2016). Mycotoxin Detection. Curr. Opin. Biotechnol..

[3] Logrieco A., Moretti A., Perrone G., Mulè G. (2007). Biodiversity of Complexes of Mycotoxigenic Fungal Species Associated with Fusarium Ear Rot of Maize and Aspergillus Rot of Grape. Int. J. Food Microbiol..

[4] Patriarca A. (2016). *Alternaria* in Food Products. Curr. Opin. Food Sci..

[5] Frisvad J.C., Hubka V., Ezekiel C.N., Hong S.-B., Nováková A., Chen A.J., Arzanlou M., Larsen T.O., Sklenář F., Mahakarnchanakul W. (2019). Taxonomy of *Aspergillus* Section *Flavi* and Their Production of Aflatoxins, Ochratoxins and Other Mycotoxins. Stud. Mycol..

[6] Zakaria L. (2024). An Overview of *Aspergillus* Species Associated with Plant Diseases. Pathogens.

[7] Pushparaj K., Meyyazhagan A., Pappuswamy M., Mousavi Khaneghah A., Liu W.-C., Balasubramanian B. (2023). Occurrence, Identification, and Decontamination of Potential Mycotoxins in Fruits and Fruit By-Products. Food Front..

[8] IARC Working Group on the Evaluation of Carcinogenic Risks to Humans (2013). Some Chemicals Present in Industrial and Consumer Products, Food and Drinking-Water.

[9] European Commission (2023). Commission Regulation (EU) 2023/915 of 25 April 2023 on Maximum Levels for Certain Contaminants in Food and Repealing Regulation (EC) No 1881/2006. Off. J. Eur. Union.

[10] European Commission (2024). Commission Regulation (EU) 2024/1022 of 8 April 2024 Amending Regulation (EU) 2023/915 as Regards Maximum Levels of Deoxynivalenol in Food. Off. J. Eur. Union.

[11] Savic I., Lucev M., Nikolić A., Vico I., Mladenović-Drinić S., Duduk N., Stanković S. (2021). The Morphological and Molecular Identification of *Fusarium verticillioides* Causing Fusariosis on Wheat Grain. Genetika.

[12] Alshannaq A., Yu J.H. (2017). Occurrence, Toxicity, and Analysis of Major Mycotoxins in Food. Int. J. Environ. Res. Public Health.

[13] Hayat A., Paniel A., Rhouati A., Marty J.-L., Barthelmebs L. (2012). Recent Advances in Ochratoxin A-Producing Fungi Detection Based on PCR Methods and Ochratoxin A Analysis in Food Matrices. Food Control.

[14] Sdogati S., Pecorelli I., Orsini S., Pacini T., Bibi R., Caporali A., Verdini E., Smaoui S., Agriopoulou S., D’Amore T. (2026). *Alternaria* Toxins in Tomato Products: From Analytical Advances to Risk Assessment and Regulatory Needs. Curr. Res. Food Sci..

[15] Bansal M., Saifi I.J., Dev I., Sonkar A.K., Dixit S., Singh S.P., Ansari K.M. (2021). Occurrence of Alternariol and Alternariolmonomethyl Ether in Edible Oils: Their Thermal Stability and Intake Assessment in State of Uttar Pradesh, India. J. Food Sci..

[16] Kustrzeba-Wójcicka I., Siwak E., Terlecki G., Wolańczyk-Mędrala A., Mędrala W. (2014). *Alternaria alternata* and Its Allergens: A Comprehensive Review. Clin. Rev. Allergy Immunol..

[17] Xue M., Qu Z., Moretti A., Logrieco A., Chu H., Zhang Q., Sun C., Ren X., Cui L., Chen Q. (2025). Aspergillus Mycotoxins: The Major Food Contaminants. Adv. Sci..

[18] Moshcheva A.G., Shykhalieva F.D., Galvidis I.A., Melekhin A.O., Burkin M.A. (2025). Occurrence of *Alternaria* Mycotoxins and Aflatoxins Contamination in Vegetable Oils by Enzyme Immunoassay Study. One Health Adv..

[19] Kaireva G., Kazxbaxexa S., Korabaxexa S., Ismagulaxa E., Tursunoxa A., Almakhanoxa S., Turuspekexa S., Askarova M., Gritsenko D. (2026). Morphological, Molecular, and Pathogenic Characterization of *Alternaria alternata* Isolates from Apple. Horticulturae.

[20] Vinogradova S.A., Kiselev K.V., Suprun A.R. (2026). An Overview of the *Alternaria* Genus: Ecology, Pathogenicity and Importance for Agriculture and Human Health. J. Fungi.

[21] Ahmad T., Xing F., Cao C., Liu Y. (2024). Characterization and Toxicological Potential of *Alternaria alternata* Associated with Post-Harvest Fruit Rot of *Prunus avium* in China. Front. Microbiol..

[22] Owolabi I.O., Kolawole O., Jantarabut P., Elliott C.T., Petchkongkaew A. (2022). The Importance and Mitigation of Mycotoxins and Plant Toxins in Southeast Asian Fermented Foods. npj Sci. Food.

[23] (2011). EFSA Panel on Contaminants in the Food Chain (CONTAM). Scientific Opinion on the Risks for Animal and Public Health Related to the Presence of *Alternaria* Toxins in Feed and Food. EFSA J..

[24] Hamad G.M., Mehany T., Simal-Gandara J., Abou-Alella S., Esua O.J., Abdel-Wahhab M.A., Hafez E.E. (2023). A Review of Recent Innovative Strategies for Controlling Mycotoxins in Foods. Food Control.

[25] Berthiller F., Crews C., Dall’Asta C., De Saeger S., Haesaert G., Karlovsky P., Oswald I.P., Seefelder W., Speijers G., Stroka J. (2013). Masked Mycotoxins: A Review. Mol. Nutr. Food Res..

[26] Nešić K., Habschied K., Mastanjević K. (2023). Modified Mycotoxins and Multitoxin Contamination of Food and Feed as Major Analytical Challenges. Toxins.

[27] Zhang Z., Nie D., Fan K., Yang J., Guo W., Meng J., Zhao Z., Han Z. (2020). A Systematic Review of Plant-Conjugated Masked Mycotoxins: Occurrence, Toxicology, and Metabolism. Crit. Rev. Food Sci. Nutr..

[28] Khan R. (2024). Mycotoxins in Food: Occurrence, Health Implications, and Control Strategies—A Comprehensive Review. Toxicon.

[29] European Commission (2026). Legislation—Contaminants in Food. https://food.ec.europa.eu/food-safety/chemical-safety/contaminants/legislation_en.

[30] Rahman H.U., Yue X., Yu Q., Zhang W., Zhang Q., Li P. (2020). Current PCR-Based Methods for the Detection of Mycotoxigenic Fungi in Complex Food and Feed Matrices. World Mycotoxin J..

[31] Meskini M., Rezghi Rami M., Tavakoli R., Salami M. (2026). Molecular Markers in Diagnostics of Fungi and Fungal Mycotoxins: A Narrative Review. J. Infect. Public Health.

[32] Bretträger M., Becker T., Gastl M. (2022). Screening of Mycotoxigenic Fungi in Barley and Barley Malt (*Hordeum vulgare* L.) Using Real-Time PCR—A Comparison between Molecular Diagnostic and Culture Technique. Foods.

[33] Al-Zaban M.I., Alrokban A.H., Mahmoud M.A. (2023). Development of a Real-Time PCR and Multiplex PCR Assay for the Detection and Identification of Mycotoxigenic Fungi in Stored Maize Grains. Mycology.

[34] Edwards S.G., O’Callaghan J., Dobson A.D.W. (2002). PCR-Based Detection and Quantification of Mycotoxigenic Fungi. Mycol. Res..

[35] Shephard G.S. (2008). Determination of Mycotoxins in Human Foods. Chem. Soc. Rev..

[36] Crous P.W., Hawksworth D.L., Wingfield M.J. (2015). Identifying and Naming Plant-Pathogenic Fungi: Past, Present, and Future. Annu. Rev. Phytopathol..

[37] Samson R.A., Visagie C.M., Houbraken J., Hong S.B., Hubka V., Klaassen C.H.W., Perrone G., Seifert K.A., Susca A., Tanney J.B. (2014). Phylogeny, Identification and Nomenclature of the Genus *Aspergillus*. Stud. Mycol..

[38] Senayake I.C., Rathnayaka A.R., Marasinghe D.S., Calabon M.S., Gentekaki E., Lee H.B., Hurdeal V.G., Pem D., Dissanayake L.S., Wijesinghe S.N. (2020). Morphological Approaches in Studying Fungi: Collection, Examination, Isolation, Sporulation and Preservation. Mycosphere.

[39] Tournas V., Stack M.E., Mislivec P.B., Koch H.A., Bandler R. (2001). Yeasts, Molds and Mycotoxins. Bacteriological Analytical Manual.

[40] Venbrux M., Crauwels S., Rediers H. (2023). Current and Emerging Trends in Techniques for Plant Pathogen Detection. Front. Plant Sci..

[41] Lucev M., Nikolić A., Jaukovic M., Savic I., Petrovic T., Bagi F., Stanković S. (2018). Differentiation between *Aspergillus flavus* and *Aspergillus parasiticus* Isolates Originated from Wheat. Genetika.

[42] Alkuwari A., Hassan Z.U., Zeidan R., Al-Thani R., Jaoua S. (2022). Occurrence of Mycotoxins and Toxigenic Fungi in Cereals and Application of Yeast Volatiles for Their Biological Control. Toxins.

[43] Griffith G.W., Easton G.L., Detheridge A., Roderick K., Edwards A., Worgan H.J., Nicholson J., Perkins W.T. (2007). Copper Deficiency in Potato Dextrose Agar Causes Reduced Pigmentation in Cultures of Various Fungi. FEMS Microbiol. Lett..

[44] Zhong J., Zhang S., Huang W., Cheng J., Li X., Wang S., Zhang T., Sui G. (2026). Multiplex On-Chip Detection of *Aspergillus* by Integrated Ultrasonication-Based Bead Beating Lysis and Magnetic Beads Direct Amplification. Front. Bioeng. Biotechnol..

[45] Khan R., Mohamad Ghazali F., Mahyudin N.A., Samsudin N.I.P. (2020). Morphological Characterization and Determination of Aflatoxigenic and Non-Aflatoxigenic *Aspergillus flavus* Isolated from Sweet Corn Kernels and Soil in Malaysia. Agriculture.

[46] Thathana M.G., Murage H., Abia A.L.K., Pillay M. (2017). Morphological Characterization and Determination of Aflatoxin-Production Potentials of *Aspergillus flavus* Isolated from Maize and Soil in Kenya. Agriculture.

[47] Diba K., Kordbacheh P., Mirhendi S.H., Rezaie S., Mahmoudi M. (2007). Identification of *Aspergillus* Species Using Morphological Characteristics. Pak. J. Med. Sci..

[48] Gangaraj R., Nagaraja A., Gaba S., Das A., Prameeladevi T., Debbarma R., Choudhary S.P., Kumari A., Kamil D. (2022). Occurrence, Identification and Pathogenicity of *Fusarium* Species Associated with Guava Wilt Disease in India. Arch. Phytopathol. Plant Prot..

[49] Tang C., Xu Q., Zhao M., Wang X., Kang Z. (2018). Understanding the Lifestyles and Pathogenicity Mechanisms of Obligate Biotrophic Fungi in Wheat: The Emerging Genomics Era. Crop J..

[50] Wijayawardene N.N., Bahram M., Sánchez-Castro I., Dai D.Q., Ariyawansa K.G.S.U., Jayalal U., Suwannarach N., Tedersoo L. (2021). Current Insight into Culture-Dependent and Culture-Independent Methods in Discovering Ascomycetous Taxa. J. Fungi.

[51] Wickes B.L., Romanelli A.M. (2020). Diagnostic Mycology: Xtreme Challenges. J. Clin. Microbiol..

[52] Parlascino R., Conti Taguali S., Riolo M., Moricca S., Bakonyi J., Ezra D., Benigno A., La Spada F., Pane A., Cacciola S.O. (2025). Unveiling the Invisible Threat: Cutting-Edge Methods for Detecting Fungal and Oomycete Plant Pathogens and Mycotoxins. Physiol. Mol. Plant Pathol..

[53] Yeni F., Acar S., Polat Ö.G., Soyer Y., Alpas H. (2014). Rapid and standardized methods for detection of foodborne pathogens and mycotoxins on fresh produce. Food Control.

[54] Buslyk T.V., Rosalovsky V.P., Salyha Y.T. (2022). PCR-Based Detection and Quantification of Mycotoxin-Producing Fungi. Cytol. Genet..

[55] Scheu P.M., Berghof K., Stahl U. (1998). Detection of Pathogenic and Spoilage Micro-Organisms in Food with the Polymerase Chain Reaction. Food Microbiol..

[56] Konstantinova P., Bonants P.J.M., van Gent-Pelzer M.P.E., van der Zouwen P., van den Bulk R. (2002). Development of Specific Primers for Detection and Identification of *Alternaria* spp. in Carrot Material by PCR and Comparison with Blotter and Plating Assays. Mycol. Res..

[57] Hong S.G., Cramer R.A., Lawrence C.B., Pryor B.M. (2005). Alt a 1 Allergen Homologs from *Alternaria* and Related Taxa: Analysis of Phylogenetic Content and Secondary Structure. Fungal Genet. Biol..

[58] Pavón M.Á., González I., Pegels N., Martín R., García T. (2010). PCR detection and identification of *Alternaria* species-groups in processed foods based on the genetic marker Alt a 1. Food Control.

[59] Chou H.-H., Wu W.-S. (2002). Phylogenetic Analysis of Internal Transcribed Spacer Regions of the Genus *Alternaria*, and the Significance of Filament-Beaked Conidia. Mycol. Res..

[60] Rashmi R., Ramana M.V., Shylaja R., Uppalapati S.R., Murali H.S., Batra H.V. (2013). Evaluation of a Multiplex PCR Assay for Concurrent Detection of Four Major Mycotoxigenic Fungi from Foods. J. Appl. Microbiol..

[61] Krnjaja V., Stanković S., Obradović A., Petrović T., Mandić V., Bijelić Z., Božić M. (2018). Trichothecene Genotypes of *Fusarium graminearum* Populations Isolated from Winter Wheat Crops in Serbia. Toxins.

[62] Pasquali M., Beyer M., Logrieco A., Audenaert K., Balmas V., Basler R., Boutigny A.-L., Chrpová J., Czembor E., Gagkaeva T. (2016). A European Database of *Fusarium graminearum* and *F. culmorum* Trichothecene Genotypes. Front. Microbiol..

[63] Okechukwu V.O., Adelusi O.A., Fagbohun T.R., Okonkwo O.J., Njobeh P.B. (2026). Mycotoxin Detection in Food and Feed: Bridging Conventional Analytical Methods with Emerging Sensor-Based Technologies. Mycotoxin Res..

[64] Wacoo A.P., Wendiro D., Vuzi P.C., Hawumba J.F. (2014). Methods for Detection of Aflatoxins in Agricultural Food Crops. J. Appl. Chem..

[65] Rocha L.F., Srour A.Y., Pimentel M., Subedi A., Bond J.P., Fakhoury A., Ammar H.A. (2023). A Panel of qPCR Assays to Detect and Quantify Soybean Soil-Borne Pathogens. Lett. Appl. Microbiol..

[66] Chen L., Lü G., Yang S., Gong B., Lu Y., Wu X., Li J., Gao H. (2024). Advances in the Detection Technology of Vegetable Soil-Borne Fungi and Bacteria. Front. Microbiol..

[67] Deng J., Zhao M., Jiang H. (2025). Research Progress in the Detection of Mycotoxins in Cereals and Their Products by Vibrational Spectroscopy. Foods.

[68] Bartolić D., Stanković M., Mutavdžić D., Stanković S., Jovanović D., Radotić K. (2018). Multivariate Curve Resolution—Alternate Least Square Analysis of Excitation-Emission Matrices for Maize Flour Contaminated with Aflatoxin B1. J. Fluoresc..

[69] Bartolić D., Mutavdžić D., Carstensen J., Stanković S., Lucev M., Krstović S., Radotić K. (2022). Fluorescence Spectroscopy and Multispectral Imaging for Fingerprinting of Aflatoxin-B1 Contaminated (*Zea mays* L.) Seeds: A Preliminary Study. Sci. Rep..

[70] Zhou S., Xu L., Kuang H., Xiao J., Xu C. (2020). Immunoassays for Rapid Mycotoxin Detection: State of the Art. Analyst.

[71] Rahman H.U., Yue X., Yu Q., Xie H., Zhang W., Zhang Q., Li P. (2019). Specific Antigen-Based and Emerging Detection Technologies of Mycotoxins. J. Sci. Food Agric..

[72] Chen M., Qileng A., Liang H., Lei H., Liu W., Liu Y. (2023). Advances in Immunoassay-Based Strategies for Mycotoxin Detection in Food: From Single-Mode Immunosensors to Dual-Mode Immunosensors. Compr. Rev. Food Sci. Food Saf..

[73] Arora D.K., Das S., Sukumar M. (2013). ELISA-Based Identification and Detection of Microbes. Analyzing Microbes.

[74] Green B.J., Schmechel D., Tovey E.R. (2005). Detection of Aerosolized *Alternaria alternata* Conidia, Hyphae, and Fragments by Using a Novel Double-Immunostaining Technique. Clin. Diagn. Lab. Immunol..

[75] Osibe D.A., Lei S., Wang B., Jin C., Fang W. (2020). Cell Wall Polysaccharides from Pathogenic Fungi for Diagnosis of Fungal Infectious Disease. Mycoses.

[76] Wang Y., Zhang C., Wang J., Knopp D. (2022). Recent Progress in Rapid Determination of Mycotoxins Based on Emerging Biorecognition Molecules: A Review. Toxins.

[77] Ong J.Y., Pike A., Tan L.L. (2021). Recent Advances in Conventional Methods and Electrochemical Aptasensors for Mycotoxin Detection. Foods.

[78] Li Y., Zhang J., Mao X., Wu Y., Liu G., Song L., Li Y., Yang J., You Y., Cao X. (2017). High-Sensitivity Chemiluminescent Immunoassay Investigation and Application for the Detection of T-2 Toxin and Major Metabolite HT-2 Toxin. J. Sci. Food Agric..

[79] Wang T., Li P., Zhang Q., Zhang W., Zhang Z., Wang T., He T. (2017). Determination of *Aspergillus* Pathogens in Agricultural Products by a Specific Nanobody-Polyclonal Antibody Sandwich ELISA. Sci. Rep..

[80] Agriopoulou S., Stamatelopoulou E., Varzakas T. (2020). Advances in Analysis and Detection of Major Mycotoxins in Foods. Foods.

[81] Oplatowska-Stachowiak M., Kleintjens T., Sajic N., Haasnoot W., Campbell K., Elliott C.T., Salden M. (2017). T-2 Toxin/HT-2 Toxin and Ochratoxin A ELISAs Development and In-House Validation in Food in Accordance with the Commission Regulation (EU) No 519/2014. Toxins.

[82] Sarmah D., Dhanabalan S.P., Johnson I., Sekar C., Mary X.A., Karthikeyan M. (2025). Diagnostic Tools for Detection of Pathogens and Diseases in Plantation Crops. J. Microbiol. Methods.

[83] Xue S., Li H.-P., Zhang J.-B., Liu J.-L., Hu Z.-Q., Gong A.-D., Huang T., Liao Y.-C. (2013). Chicken Single-Chain Antibody Fused to Alkaline Phosphatase Detects *Aspergillus* Pathogens and Their Presence in Natural Samples by Direct Sandwich Enzyme-Linked Immunosorbent Assay. Anal. Chem..

[84] Ansari S., Mousavi A., Safarnejad M.R., Farrokhi N., Alavi S.M., Schillberg S., Nölke G. (2021). Selection and Characterization of Two Monoclonal Antibodies Specific for the *Aspergillus flavus* Major Antigenic Cell Wall Protein Aflmp1. Fungal Biol..

[85] Park J.W., Shon D.H., Kim Y.B. (2003). Application of an Enzyme-Linked Immunosorbent Assay for Detecting Mold Contamination in Agricultural Commodities and Comparison with Conventional Assays. Food Agric. Immunol..

[86] Boshra M.H., El-Housseiny G.S., Farag M.M.S., Aboshanab K.M. (2023). Evaluation of ELISA and Immunoaffinity Fluorometric Analytical Tools of Four Mycotoxins in Various Food Categories. AMB Express.

[87] Hafez E., Abd El-Aziz N.M., Darwish A.M.G., Shehata M.G., Ibrahim A.A., Elframawy A.M., Badr A.N. (2021). Validation of New ELISA Technique for Detection of Aflatoxin B1 Contamination in Food Products versus HPLC and VICAM. Toxins.

[88] Omar S.S., Haddad M.A., Parisi S. (2020). Validation of HPLC and Enzyme-Linked Immunosorbent Assay (ELISA) Techniques for Detection and Quantification of Aflatoxins in Different Food Samples. Foods.

[89] Lei H., Wang Z., Eremin S.A., Liu Z. (2022). Application of Antibody and Immunoassay for Food Safety. Foods.

[90] Kagdi J.H., Mehta V.N., Jha S., Park T.J., Basu H., Kailasa S.K. (2026). Progress of Lateral Flow Assays for the Detection of Molecular and Microbial Species: From Basic Formats to Microfluidics, CRISPR and Artificial Intelligence. Analyst.

[91] Kakkar S., Gupta P., Singh Yadav S.P., Raj D., Singh G., Chauhan S., Mishra M.K., Martín-Ortega E., Chiussi S., Kant K. (2024). Lateral Flow Assays: Progress and Evolution of Recent Trends in Point-of-Care Applications. Mater. Today Bio.

[92] Zhao S., Wang S., Zhang S., Liu J., Dong Y. (2018). State of the Art: Lateral Flow Assay (LFA) Biosensor for On-Site Rapid Detection. Chin. Chem. Lett..

[93] Contreras Alvarez L.A., Lazo Jara M.D., Campos F.V., de Oliveira J.P., Guimarães M.C.C. (2024). Barcode-Style Lateral Flow Immunochromatographic Strip for the Semi-Quantitative Detection of Ochratoxin A in Coffee Samples. Food Addit. Contam. Part A.

[94] Wakeham A.J., Keane G., Kennedy R. (2016). Field Evaluation of a Competitive Lateral-Flow Assay for Detection of *Alternaria brassicae* in Vegetable Brassica Crops. Plant Dis..

[95] Liang M., Hu X., Du W., Tang X., Zhang Q., Li P. (2025). Catalytic Boost in Dual-Active Sites Hierarchical Hollow Nanozymes: An Enhanced Robust and Sensitive Immunochromatographic Assay for *Aspergillus flavus*. Biosens. Bioelectron..

[96] Liang M., Cai X., Gao Y., Yan H., Fu J., Tang X., Zhang Q., Li P. (2022). A Versatile Nanozyme Integrated Colorimetric and Photothermal Lateral Flow Immunoassay for Highly Sensitive and Reliable *Aspergillus flavus* Detection. Biosens. Bioelectron..

[97] Adunphatcharaphon S., Kolawole O., Sooksimuang T., Panchan W., Wasuthep W., Petdum A., Pichayawaytin G., Jintamethasawat R., Doljirapisit N., Somboonkaew A. (2024). A Multiplex Microarray Lateral Flow Immunoassay Device for Simultaneous Determination of Five Mycotoxins in Rice. npj Sci. Food.

[98] Xing Y., Yang S., Fan L., Hu X., Liu S., Wang Y., Sun Y. (2025). A Time-Resolved Fluorescent Lateral Flow Immunoassay for the Rapid and Ultra-Sensitive Detection of AFB1 in Peanuts and Maize. Foods.

[99] Li P., Zhang Q., Zhang W., Zhang J., Chen X., Jiang J., Xie L., Zhang D. (2009). Development of a Class-Specific Monoclonal Antibody-Based ELISA for Aflatoxins in Peanut. Food Chem..

[100] Beloglazova N.V., Graniczkowska K., Ediage E.N., Averkieva O., De Saeger S. (2017). Sensitive Flow-through Immunoassay for Rapid Multiplex Determination of Cereal-borne Mycotoxins in Feed and Feed Ingredients. J. Agric. Food Chem..

[101] Li T., Jo E.J., Kim M.G. (2012). A Label-Free Fluorescence Immunoassay System for the Sensitive Detection of the Mycotoxin Ochratoxin A. Chem. Commun..

[102] Roda A., Mirasoli M., Michelini E., Di Fusco M., Zangheri M., Cevenini L., Simoni P. (2016). Progress in Chemical Luminescence-Based Biosensors: A Critical Review. Biosens. Bioelectron..

[103] Yang Y., Ren M.-Y., Xu X.-G., Han Y., Zhao X., Li C.-H., Zhao Z.-L. (2024). Recent Advances in Simultaneous Detection Strategies for Multi-Mycotoxins in Foods. Crit. Rev. Food Sci. Nutr..

[104] Liu X., Li J., Chen R., Xie X., Mao F., Sun Z., He Z., Cao H., Zhang S., Liu X. (2024). Colorimetric and Chemiluminescent Enzyme Immunoassays Based on the Alkaline Phosphatase-Tagged Single-Chain Variable Fragment Fusion Tracer for Detecting Zearalenone in Agro-Products. Food Chem..

[105] Wang Y., Jia K., Lin J. (2024). Optical Biosensors for the Detection of Foodborne Pathogens: Recent Development and Future Prospects. TrAC Trends Anal. Chem..

[106] Wu K., He X., Wang J., Pan T., He R., Kong F., Cao Z., Ju F., Huang Z., Nie L. (2022). Recent Progress of Microfluidic Chips in Immunoassay. Front. Bioeng. Biotechnol..

[107] Li R., Meng C., Wen Y., Fu W., He P. (2019). Fluorometric Lateral Flow Immunoassay for Simultaneous Determination of Three Mycotoxins (Aflatoxin B1, Zearalenone and Deoxynivalenol) Using Quantum Dot Microbeads. Microchim. Acta.

[108] Janik-Karpińska E., Ceremuga M., Niemcewicz M., Podogrocki M., Stela M., Cichoń N., Bijak M. (2022). Immunosensors—The Future of Pathogen Real-Time Detection. Sensors.

[109] Mollarasouli F., Kurbanoglu S., Ozkan S.A. (2019). The Role of Electrochemical Immunosensors in Clinical Analysis. Biosensors.

[110] Szelenberger R., Cichoń N., Zajączkowski W., Bijak M. (2024). Application of Biosensors for the Detection of Mycotoxins for the Improvement of Food Safety. Toxins.

[111] Cancelliere R., Paialunga E., Grattagliano A., Micheli L. (2024). Label-Free Electrochemical Immunosensors: A Practical Guide. TrAC Trends Anal. Chem..

[112] Jia M., Liao X., Fang L., Jia B., Liu M., Li D., Zhou L., Kong W. (2021). Recent Advances on Immunosensors for Mycotoxins in Foods and Other Commodities. TrAC Trends Anal. Chem..

[113] Patel H. (2025). Immunosensors for Mycotoxin Detection: Techniques, Applications and Future Directions. Mycotoxin Res..

[114] Wei T., Ren P., Huang L., Ouyang Z., Wang Z., Kong X., Li T., Yin Y., Wu Y., He Q. (2019). Simultaneous Detection of Aflatoxin B1, Ochratoxin A, Zearalenone and Deoxynivalenol in Corn and Wheat Using Surface Plasmon Resonance. Food Chem..

[115] Yuan J., Deng D., Lauren D.R., Aguilar M.I., Wu Y. (2009). Surface Plasmon Resonance Biosensor for the Detection of Ochratoxin A in Cereals and Beverages. Anal. Chim. Acta.

[116] Bonel L., Vidal J.C., Duato P., Castillo J.R. (2010). Ochratoxin A Nanostructured Electrochemical Immunosensors Based on Polyclonal Antibodies and Gold Nanoparticles Coupled to the Antigen. Anal. Methods.

[117] Vidal J.C., Bonel L., Duato P., Castillo J.R. (2011). Improved Electrochemical Competitive Immunosensor for Ochratoxin A with a Biotinylated Monoclonal Antibody Capture Probe and Colloidal Gold Nanostructuring. Anal. Methods.

[118] Chen L., Jiang J., Shen G., Yu R. (2015). A Label-Free Electrochemical Impedance Immunosensor for the Sensitive Detection of Aflatoxin B1. Anal. Methods.

[119] Lee J., Kim S., Chung H.Y., Kang A., Kim S., Hwang H., Yang S.I., Yun W.S. (2021). Electrochemical Microgap Immunosensors for Selective Detection of Pathogenic *Aspergillus niger*. J. Hazard. Mater..

[120] Pogner C., Gorfer M., Raulf M., Strauss J., Sander I. (2025). Quantification of Airborne Fungal Antigens by ELISA and Comparison to Molecular Biological and Classical Methods. Appl. Environ. Microbiol..

[121] Homola J. (2008). Surface Plasmon Resonance Sensors for Detection of Chemical and Biological Species. Chem. Rev..

[122] Kuca K., Kumar H., Kala D. (2025). Ensuring Wellness: Immunosensors Monitoring Food Quality, and Pathogenicity Risks.

[123] Hou Y., Jia B., Sheng P., Liao X., Shi L., Fang L., Zhou L., Kong W. (2022). Aptasensors for Mycotoxins in Foods: Recent Advances and Future Trends. Compr. Rev. Food Sci. Food Saf..

[124] Fan Y., Li J., Amin K., Yu H., Yang H., Guo Z., Liu J. (2023). Advances in Aptamers, and Application of Mycotoxins Detection: A Review. Food Res. Int..

[125] Kumar A.Y.V.V.A., Renuka R.M., Achuth J., Venkataramana M., Ushakiranmayi M., Sudhakar P. (2018). Development of Hybrid IgG-Aptamer Sandwich Immunoassay Platform for Aflatoxin B1 Detection and Its Evaluation onto Various Field Samples. Front. Pharmacol..

[126] Torres-Chavolla E., Alocilja E.C. (2009). Aptasensors for Detection of Microbial and Viral Pathogens. Biosens. Bioelectron..

[127] Ellington A.D., Szostak J.W. (1990). In Vitro Selection of RNA Molecules That Bind Specific Ligands. Nature.

[128] Tuerk C., Gold L. (1990). Systematic Evolution of Ligands by Exponential Enrichment: RNA Ligands to Bacteriophage T4 DNA Polymerase. Science.

[129] Cheng S., Xu J., Gao P., Cai J., Cao Y., Hong Y., Ding H., Chen H., Liu X., Zhang G. (2026). Advancements in Aptasensors Development for the Detection of Zearalenone. Front. Nutr..

[130] Tavassoli M., Khezerlou A., Khalilzadeh B., Ehsani A., Kazemian H. (2023). Aptamer-Modified Metal Organic Frameworks for Measurement of Food Contaminants: A Review. Microchim. Acta.

[131] Parihar A., Choudhary N.K., Sharma P., Khan R. (2023). MXene-Based Aptasensor for the Detection of Aflatoxin in Food and Agricultural Products. Environ. Pollut..

[132] Dadmehr M., Shahi S.C., Malekkiani M., Korouzhdehi B., Tavassoli A. (2023). A Stem-Loop-Like Aptasensor for Sensitive Detection of Aflatoxin Based on Graphene Oxide/AuNPs Nanocomposite Platform. Food Chem..

[133] Fang P., Qu H., Mao Y., Zheng L. (2023). Aptamers for Mycotoxin Recognition in Food: Recent Advances and Future Considerations. Adv. Agrochem..

[134] Gao S., Zhou R., Zhang D., Zheng X., El-Seedi H.R., Chen S., Niu L., Li X., Guo Z., Zou X. (2024). Magnetic Nanoparticle-Based Immunosensors and Aptasensors for Mycotoxin Detection in Foodstuffs: An Update. Compr. Rev. Food Sci. Food Saf..

[135] Seo J.-W., Kim J.Y., Kim D.H., Oh J.-J., Kim Y.J., Kim G.-H. (2021). Selection and Characterization of Toxic *Aspergillus* Spore-Specific DNA Aptamer Using Spore-SELEX. RSC Adv..

[136] Low S.Y., Hill J.E., Peccia J. (2009). DNA Aptamers Bind Specifically and Selectively to (1→3)-β-D-Glucans. Biochem. Biophys. Res. Commun..

[137] Yan X., Chen H., Du G., Guo Q., Yuan Y., Yue T. (2022). Recent Trends in Fluorescent Aptasensors for Mycotoxin Detection in Food: Principles, Constituted Elements, Types, and Applications. Food Front..

[138] Wang C., Zhao X., Huang X., Xu F., Gu C., Yu S., Zhang X., Qian J. (2024). Simultaneous Detection of Multiple Mycotoxins Using MXene-Based Electrochemical Aptasensor Array and a Self-Developed Multi-Channel Portable Device. Talanta.

[139] Zhang X., Zhang J., Jiang Y., Zheng Q., Hua M.Z., Lu X., Cao J. (2026). Dual-Mode PEC-Colorimetric Aptasensor for Sensitive Aflatoxin B1 Detection via a BiVO4/Ni3(HHTP)2 Heterojunction and PdAuCu Nanoenzyme Amplification. Food Res. Int..

[140] Li H., Guo L., Liu L., Xu C., Kuang H., Qu A. (2026). Recent Advances in Immunoassays for Biotoxin Detection in Food Samples. TrAC Trends Anal. Chem..

[141] Li W., Xu Z., He Q., Pan J., Zhang Y., El-Sheikh E.-S.A., Hammock B.D., Li D. (2025). Nanobody-Based Immunoassays for the Detection of Food Hazards—A Review. Biosensors.

[142] Nawaz M.A.H., Rauf S., Hayat A., Catanante G., Raza R., Marty J.-L., Wong Y.-C., Lewis R.J. (2017). Determination of Mycotoxins in Food. Analysis of Food Toxins and Toxicants.

[143] Bian Y., Zhang Y., Zhou Y., Wei B., Feng X. (2023). Recent Insights into Sample Pretreatment Methods for Mycotoxins in Different Food Matrices: A Critical Review on Novel Materials. Toxins.

[144] Chen X., Abdallah M.F., Landschoot S., Audenaert K., De Saeger S., Chen X., Rajkovic A. (2023). *Aspergillus flavus* and *Fusarium verticillioides* and Their Main Mycotoxins: Global Distribution and Scenarios of Interactions in Maize. Toxins.

[145] Schiro G., Verch G., Grimm V., Müller M.E.H. (2018). *Alternaria* and *Fusarium* Fungi: Differences in Distribution and Spore Deposition in a Topographically Heterogeneous Wheat Field. J. Fungi.

[146] Alisaac E., Behmann J., Rathgeb A., Karlovsky P., Dehne H.W., Mahlein A.K. (2019). Assessment of *Fusarium* Infection and Mycotoxin Contamination of Wheat Kernels and Flour Using Hyperspectral Imaging. Toxins.

[147] Chu X., Wang W., Yoon S.-C., Ni X., Heitschmidt G.W. (2017). Detection of Aflatoxin B1 (AFB1) in Individual Maize Kernels Using Short Wave Infrared (SWIR) Hyperspectral Imaging. Biosyst. Eng..

[148] Niessen L. (2007). PCR-Based Diagnosis and Quantification of Mycotoxin Producing Fungi. Int. J. Food Microbiol..

[149] Turner N.W., Subrahmanyam S., Piletsky S.A. (2009). Analytical Methods for Determination of Mycotoxins: A Review. Anal. Chim. Acta.

[150] Zheng M.Z., Richard J.L., Binder J. (2006). A Review of Rapid Methods for the Analysis of Mycotoxins. Mycopathologia.

[151] Free S.J. (2013). Fungal Cell Wall Organization and Biosynthesis. Adv. Genet..

[152] Gow N.A.R., Lenardon M.D. (2023). Architecture of the Dynamic Fungal Cell Wall. Nat. Rev. Microbiol..

[153] Cho H.J., Son S.H., Chen W., Son Y.E., Lee I., Yu J.H., Park H.S. (2022). Regulation of Conidiogenesis in *Aspergillus flavus*. Cells.

[154] Sanna M., Martino I., Guarnaccia V., Mezzalama M. (2023). Diversity and Pathogenicity of *Fusarium* Species Associated with Stalk and Crown Rot in Maize in Northern Italy. Plants.

[155] Mwangi M.W., Muiru W.M., Kimenju J.W. (2021). Characterisation of *Fusarium* Species Infecting Tomato in Mwea West Sub-County, Kirinyaga County, Kenya. Can. J. Plant Pathol..

[156] Tymon L.S., Peever T.L., Johnson D.A. (2016). Identification and Enumeration of Small-Spored *Alternaria* Species Associated with Potato in the U.S. Northwest. Plant Dis..

[157] Latgé J.P. (2007). The Cell Wall: A Carbohydrate Armour for the Fungal Cell. Mol. Microbiol..

[158] Fredricks D.N., Smith C., Meier A. (2005). Comparison of Six DNA Extraction Methods for Recovery of Fungal DNA from Fungal Cultures and Clinical Specimens. J. Clin. Microbiol..

[159] Haugland R.A., Heckman J.L., Wymer L.J. (1999). Evaluation of different methods for the extraction of DNA from fungal conidia by quantitative competitive PCR analysis. J. Microbiol. Methods.

[160] Cordero R.J.B., Casadevall A. (2017). Functions of Fungal Melanin beyond Virulence. Fungal Biol. Rev..

[161] Aimanianda V., Bayry J., Bozza S., Kniemeyer O., Perruccio K., Elluru S.R., Clavaud C., Paris S., Brakhage A.A., Kaveri S.V. (2009). Surface Hydrophobin Prevents Immune Recognition of Airborne Fungal Spores. Nature.

[162] Kwon-Chung K.J., Sugui J.A. (2013). *Aspergillus fumigatus*—What Makes the Species a Ubiquitous Human Fungal Pathogen?. PLoS Pathog..

[163] Dagenais T.R.T., Keller N.P. (2009). Pathogenesis of *Aspergillus fumigatus* in Invasive Aspergillosis. Clin. Microbiol. Rev..

[164] Karakousis A., Tan L., Ellis D., Alexiou H., Wormald P.J. (2006). An Assessment of the Efficiency of Fungal DNA Extraction Methods for Maximizing the Detection of Medically Important Fungi Using PCR. J. Microbiol. Methods.

[165] Molinié A., Faucet V., Castegnaro M., Pfohl-Leszkowicz A. (2005). Analysis of Some Breakfast Cereals on the French Market for Their Contents of Ochratoxin A, Citrinin and Fumonisin B1: Development of a Method for Simultaneous Extraction of Ochratoxin A and Citrinin. Food Chem..

[166] Ranathunga R.A.A., Suwannaporn P. (2022). Young Cereal Grains as a New Source of Healthy and Hypoallergenic Foods: A Review. J. Food Sci. Technol..

[167] Garutti M., Nevola G., Mazzeo R., Cucciniello L., Totaro F., Bertuzzi C.A., Caccialanza R., Pedrazzoli P., Puglisi F. (2022). The Impact of Cereal Grain Composition on the Health and Disease Outcomes. Front. Nutr..

[168] Patra A., Abdullah S., Pradhan R.C. (2022). Review on the Extraction of Bioactive Compounds and Characterization of Fruit Industry By-Products. Bioresour. Bioprocess..

[169] Hu W., Zhang X., Shen Y., Meng X., Wu Y., Tong P., Li X., Chen H., Gao J. (2024). Quantifying Allergenic Proteins Using Antibody-Based Methods or Liquid Chromatography–Mass Spectrometry/Mass Spectrometry: A Review about the Influence of Food Matrix, Extraction, and Sample Preparation. Compr. Rev. Food Sci. Food Saf..

[170] Hu W., Xiong Z., Meng X., Wu Y., Tong P., Gao J., Li X., Chen H. (2024). Lipid Matrix-Specific Pretreatment Method for Enhancing the Extractability and Allergenicity Maintenance of Bovine Milk Allergens in ELISA Detection. Food Chem..

[171] Moon Y.-J., Lee S.-Y., Oh S.-W. (2022). A Review of Isothermal Amplification Methods and Food-Origin Inhibitors against Detecting Food-Borne Pathogens. Foods.

[172] Kuffel A., Gray A., Daeid N.N. (2021). Impact of Metal Ions on PCR Inhibition and RT-PCR Efficiency. Int. J. Leg. Med..

[173] Kainz D.M., Breiner B.J., Früh S.M., Hutzenlaub T., Zengerle R., Paust N. (2021). Eliminating Viscosity Bias in Lateral Flow Tests. Microsyst. Nanoeng..

[174] Deshpande K., Kanungo L. (2023). Chemiluminescence and Fluorescence Biosensors for Food Application: A Review. Sens. Actuators Rep..

[175] Schenk J.J., Becklund L.E., Carey S.J., Fabre P.P. (2023). What Is the “Modified” CTAB Protocol? Characterizing Modifications to the CTAB DNA Extraction Protocol. Appl. Plant Sci..

[176] Masoomi-Aladizgeh F., Jabbari L., Khayam Nekouei R., Aalami A., Atwell B.J., Haynes P.A. (2023). A Universal Protocol for High-Quality DNA and RNA Isolation from Diverse Plant Species. PLoS ONE.

[177] Wang J., Li W., Wang Y., Pavase T., Zhang J., Li Z., Lin H. (2021). The Influence of Pre-Treatment Methods and Matrix Effect on Sesame (*Sesamum indicum*) Sandwich ELISA Detection. Food Agric. Immunol..

[178] Li H., Liu S., Dong B., Li C., Yang H., Zhang X., Wen K., Yu X., Yu W., Shen J. (2019). Production of a Specific Monoclonal Antibody and a Sensitive Immunoassay for the Detection of Diphacinone in Biological Samples. Anal. Bioanal. Chem..

[179] Donnelly R., Elliott C., Zhang G., Baker B., Meneely J. (2022). Understanding Current Methods for Sampling of Aflatoxins in Corn and to Generate a Best Practice Framework. Toxins.

[180] Kumphanda J., Matumba L., Monjerezi M., Whitaker T.B., De Saeger S., Makun H.A. (2021). Are Sample Size and Sample Preparation for Mycotoxin Quantitation in Grain Products Getting Trivialized?. Food Control.

[181] Pan Z., Hu J., Ou Z., Lv X., Hu B., Huang J., Xiao Z. (2026). Preparation of Anti-Idiotypic Antibodies and Their Applications in Mycotoxin Detection: A Review. Anal. Methods.

[182] Liu C.-W., Bodaghi S., Vidalakis G., Tsutsui H. (2024). Quick Plant Sample Preparation Methods Using a Micro-Homogenizer for the Detection of Multiple Citrus Pathogens. Chemosensors.

[183] Quiñones K.J.O., Gentallan R.P., Timog E.B.S., Vera Cruz J.R.A., Macabecha C.G.A., Papa I.A., Coronado N.B., Bartolome M.C.B., Ceribo D.B., Madayag R.E. (2024). Liquid-Nitrogen-Free CTAB DNA Extraction Method from Silica-Dried Specimens for Next-Generation Sequencing and Assembly. MethodsX.

[184] Carey S.J., Becklund L.E., Fabre P.P., Schenk J.J. (2023). Optimizing the Lysis Step in CTAB DNA Extractions of Silica-Dried and Herbarium Leaf Tissues. Appl. Plant Sci..

[185] Langsiri N., Meyer W., Irinyi L., Worasilchai N., Pombubpa N., Wongsurawat T., Jenjaroenpun P., Luangsa-ard J.J., Chindamporn A. (2025). Optimizing Fungal DNA Extraction and Purification for Oxford Nanopore Untargeted Shotgun Metagenomic Sequencing from Simulated Hemoculture Specimens. mSystems.

[186] Ye X., Xu C., Xie T., Zhang Y., Zhao Y., Xia C., Li Z., Huang Y., Fan J., Cao H. (2023). Myxobacterial Outer Membrane β-1,6-Glucanase Induced the Cell Death of *Fusarium oxysporum* by Destroying the Cell Wall Integrity. Appl. Environ. Microbiol..

[187] Mellikeche W., Ricelli A., Casini G., Gallo M., Baser N., Colelli G., D’Onghia A.M. (2024). Development of Loop-Mediated Isothermal Amplification (LAMP) Assays for the Rapid Detection of Toxigenic *Aspergillus flavus* and *A. carbonarius* in Nuts. Int. J. Mol. Sci..

[188] Nwe M.K., Jangpromma N., Taemaitree L. (2024). Evaluation of Molecular Inhibitors of Loop-Mediated Isothermal Amplification (LAMP). Sci. Rep..

[189] Agustinho D.P., Fu Y., Menon V.K., Metcalf G.A., Treangen T.J., Sedlazeck F.J. (2024). Unveiling Microbial Diversity: Harnessing Long-Read Sequencing Technology. Nat. Methods.

[190] Masuda S., Gan P., Kiguchi Y., Anda M., Sasaki K., Shibata A., Iwasaki W., Suda W., Shirasu K. (2024). Uncovering Microbiomes of the Rice Phyllosphere Using Long-Read Metagenomic Sequencing. Commun. Biol..

[191] Yi L., Liu H., Liu Y., Aiyisi, He J., Ming L., Jirimutu (2025). Nanobody-Based Indirect Competitive ELISA for the Detection of Aflatoxin M1 in Dairy Products. Sci. Rep..

[192] Tsoulias N., Zhao M., Paraforos D.S., Argyropoulos D., Zhang Q. (2023). Hyper- and Multi-Spectral Imaging Technologies. Encyclopedia of Smart Agriculture Technologies.

[193] Facorro R., Llompart M., Dagnac T. (2020). Combined (d)SPE-QuEChERS Extraction of Mycotoxins in Mixed Feed Rations and Analysis by High Performance Liquid Chromatography–High-Resolution Mass Spectrometry. Toxins.

[194] Kralj Cigić I., Prosen H. (2009). An Overview of Conventional and Emerging Analytical Methods for the Determination of Mycotoxins. Int. J. Mol. Sci..

[195] Sulyok M., Stadler D., Steiner D., Krska R. (2020). Validation of an LC-MS/MS-Based Dilute-and-Shoot Approach for the Quantification of >500 Mycotoxins and Other Secondary Metabolites in Food Crops: Challenges and Solutions. Anal. Bioanal. Chem..

[196] Puntscher H., Kütt M.L., Skrinjar P., Mikula H., Podlech J., Fröhlich J., Marko D., Warth B. (2018). Tracking Emerging Mycotoxins in Food: Development of an LC-MS/MS Method for Free and Modified *Alternaria* Toxins. Anal. Bioanal. Chem..

[197] Paris S., Fitting C., Ramirez E., Latgé J.-P., David B. (1990). Comparison of Different Extraction Methods of *Alternaria* Allergens. J. Allergy Clin. Immunol..

[198] Debegnach F., Gregori E., Rizzo M., Pannunzi E., Grieco M.E., De Santis B. (2026). Optimizing Sample Preparation for Homogeneous Mycotoxin Distribution in Official Control Analyses. Ann. Ist. Super. Sanità.

[199] Zhao Y., Chen D., Duan H., Li P., Wu W., Wang X., Poapolathep A., Poapolathep S., Logrieco A.F., Pascale M. (2023). Sample Preparation and Mass Spectrometry for Determining Mycotoxins, Hazardous Fungi, and Their Metabolites in the Environment, Food, and Healthcare. TrAC Trends Anal. Chem..

[200] Pouris J., Kolyva F., Bratakou S., Vogiatzi C.A., Chaniotis D., Beloukas A. (2024). The Role of Fungi in Food Production and Processing. Appl. Sci..

[201] (2016). Microbiology of the Food Chain—Method Validation—Part 2: Protocol for the Validation of Alternative (Proprietary) Methods Against a Reference Method.

[202] Scharf S., Bartels A., Kondakci M., Pfeffer K., Henrich B., Haas R. (2020). Introduction of a Bead Beating Step Improves Fungal DNA Extraction from Selected Patient Specimens. Int. J. Med. Microbiol..

[203] Rhouati A., Catanante G., Nunes G., Hayat A., Marty J.-L. (2016). Label-Free Aptasensors for the Detection of Mycotoxins. Sensors.

[204] Lemma T., Wang J., Arstila K., Hytönen V.P., Toppari J.J. (2019). Identifying Yeasts Using Surface-Enhanced Raman Spectroscopy. Spectrochim. Acta Part A.

[205] Bhardwaj N., Bhardwaj S.K., Mehta J., Kim K.-H., Deep A. (2022). Advances in Electrochemical Biosensing Strategies for Mycotoxin Detection in Food. TrAC Trends Anal. Chem..

[206] McCracken K.E., Yoon J.Y. (2016). Recent Approaches for Optical Smartphone Sensing in Resource-Limited Settings: A Brief Review. Anal. Methods.

[207] Nelis J.L.D., Tsagkaris A.S., Dillon M.J., Hajslova J., Elliott C.T. (2020). Smartphone-Based Optical Assays in the Food Safety Field. TrAC Trends Anal. Chem..

[208] Zhang J., Zhang X., Zhang Y., Yang X., Guo L., Man C., Jiang Y., Zhang W., Zhang X. (2025). Emerging Biosensors Integrated with Microfluidic Devices: A Promising Analytical Tool for On-Site Detection of Mycotoxins. npj Sci. Food.

